# Hydroxypyridinone-Diamine Hybrids as Potential Neuroprotective Agents in the PC12 Cell-Line Model of Alzheimer’s Disease

**DOI:** 10.3390/ph12040162

**Published:** 2019-10-27

**Authors:** Elodie Lohou, N. André Sasaki, Agnès Boullier, Marine Duplantier, Pascal Sonnet

**Affiliations:** 1 AGIR, EA-4294, UFR of Pharmacy, Jules Verne University of Picardie, 80037 Amiens, France; elodie.lohou@u-picardie.fr (E.L.); marine.duplantier@etud.u-picardie.fr (M.D.); 2 MP3CV, EA-7517, CURS, Amiens University Hospital, 80054 Amiens, France; agnes.boullier@u-picardie.fr; 3 UFR of Medicine, Jules Verne University of Picardie, 80037 Amiens, France; 4 Department of Biochemistry, Amiens University Hospital, 80054 Amiens, France

**Keywords:** Alzheimer’s disease, AGE inhibitors, biometals, oxidative stress, vicinal diamine, hydroxypyridinone

## Abstract

There is an urgent need to propose effective treatments for Alzheimer’s disease (AD). Although the origin of the disease is poorly understood, several therapeutic options have been proposed. The new therapeutic approaches targeting biometal-mediated neurodegenerative pathways appear to be interesting ones. As a continuation of our preceding studies, two novel series of advanced glycation endproducts (AGE)/advanced lipid peroxidation endproducts (ALE) inhibitors have been developed as multifunctional scavengers. This extended work allowed us to highlight the new hydroxypyridinone-diamine hybrid **IIa-3** bearing a C4 alkyl linker between the two pharmacophores. This derivative exhibited preserved potent capacities to trap reactive carbonyl species (vicinal diamine function) as well as reactive oxygen species and transition metals (hydroxypyridinone moiety) in comparison with previously described lead compound **1**. In addition, its good predicted absorption, distribution, metabolism and excretion (ADME) properties were correlated with a better efficacy to inhibit *in vitro* methylglyoxal-induced apoptosis in neuronal-like PC12 cells. This new promising agent revealed improved druglikeness and ability to prevent biometal-mediated oxidative and carbonyl stress amplification involved in AD pathogenesis.

## 1. Introduction

The development of drugs that can either alleviate or suppress Alzheimer’s disease (AD) onset and progression continues to be an urgent healthcare challenge due to the social and economic impacts of this pathology. New therapeutic approaches targeting biometal-mediated neurodegenerative pathways appear to be promising ones. In recent years, it has been assumed that the contribution of transition metals, such as iron and copper, as AD pathogenesis initiators supersedes the amyloid *β* (A*β*) hypothesis [1,2]. In AD brains, iron and copper overload stimulates not only APP (amyloid precursor protein) cleavage by *β*-secretase but also A*β* aggregation and toxicity. Indeed, redox-active metal ion-A*β* complexes catalyze ROS (reactive oxygen species) production leading to strong oxidative damage on biomolecules [3]. Furthermore, biometals are implicated in the formation of tau-associated neurofibrillary tangles (NFTs) that constitute the second histopathological hallmarks of AD. Fe^3+^ and Cu^2+^ promote tau phosphorylation disturbing tau-microtubule assembly and stabilization. Interaction of transition metals with free tau protein results in its aggregation and their accumulation in NFTs acts as another source of oxidative stress [1,4].

The biometal-catalyzed overproduction of the hydroxyl radical (OH**^•^**) from hydrogen peroxide (H_2_O_2_) and reductants also induces a carbonyl stress exacerbation. *a*-Oxoaldehydes like methylglyoxal (MGO) are produced through Maillard reaction between glucose and nucleophilic amine functions of proteins or other oxidative degradations of glycolysis intermediates. The condensation of these RCS (reactive carbonyl species) with proteins leads to an accumulation of deleterious advanced glycation endproducts (AGE). In AD, glycated proteins are implicated in the formation of A*β* plaques and NFTs, especially via a cross-linking promotion and the stimulation of AGE/RAGE (AGE receptors) apoptotic axis [5,6]. Furthermore, oxidative damages on polyunsaturated fatty acids (PUFAs) result in the production of another neurotoxic RCS such as 4-hydroxy-2-nonenal (4-HNE) and malondialdehyde (MDA). Michael addition of nucleophilic groups of biomolecules to these *α*,*β*-unsaturated aldehydes leads to the generation of other irreversible covalent adducts, called advanced lipid peroxidation endproducts (ALE). AGE/ALE accumulation from A*β* and tau proteins was reported to take part in the biometal-mediated vicious downward redox spiral involved in AD pathogenesis [3,7]. In addition, the ALE neurodegenerative pathway was described recently as a new iron-dependent process of programmed cell death, termed ferroptosis, that appears morphologically and biochemically different from apoptosis. Indeed, iron not only stimulates lipid peroxidation as the ROS promoter but also as the lipoxygenase catalyst. In AD, 4-HNE-associated modifications of proteins that are implicated in neuronal communication, neurite outgrowth, antioxidant and energetic metabolism induce extensive ferroptotic cell death [1,2].

**Figure 1:**
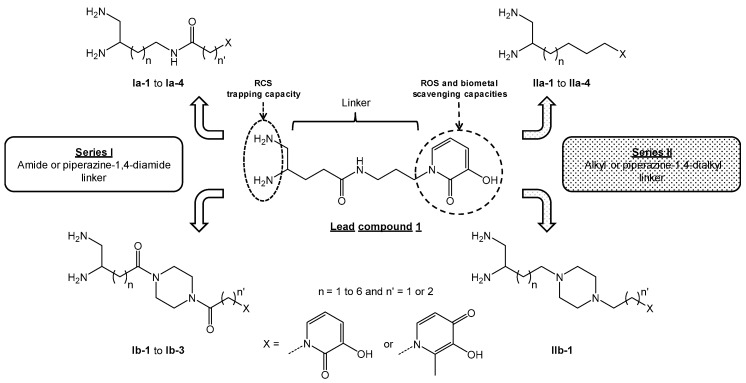
New pharmacomodulations on the linker of multifunctional advanced glycation endproducts (AGE)/advanced lipid peroxidation endproducts (ALE) inhibitor lead compound **1**.

With this AD-triggering biometal dyshomeostasis hypothesis in mind, we have previously reported the development of efficient AGE/ALE inhibitors as “multitarget-directed ligands” (MTDLs) [8,9]. These hydroxypyridinone-diamine hydrid molecules appear to be able to trap RCS (vicinal diamine function) as well as ROS and transition metals (hydroxypyridinone moiety) simultaneously. As a continuation of our efforts, extended pharmacomodulations have been realized to expand an efficacy screening and, in particular, to improve absorption, distribution, metabolism and excretion (ADME) profile of the lead compound 1, and its capacity to cross the blood brain barrier. In this paper, we report the synthesis of two novel series of derivatives bearing various suitable linkers between the two pharmacophores: i) the series **I** presenting an amide (**Ia**) or piperazine-1,4-diamide (**Ib**) spacer, and ii) the series **II** possessing an alkyl (**IIa**) or piperazine-1,4-dialkyl (**IIb**) spacer (Figure 1). We also describe their preserved scavenging capacities and a QikProp-prediction of their ADME properties. In addition, the evaluations of their cytotoxicity and their protective effect against *in vitro* MGO-induced apoptosis in the model AD cell-line PC12 are presented.

## 2. Results and Discussion

### 2.1. Chemical Synthesis

As shown in Table 1 and Figure 1, our previously described synthetic strategy based on a key pseudopeptidic coupling step between diamine building blocks **2–6** and hydroxypyridinone (HOPO) ligands **9–10** was first extended to allow a decrease in size of the amide or piperazine-1,4-diamide linker and afforded the derivatives **Ia-1** to **Ia-4** or **Ib-1** to **Ib-3** respectively [8]. Moreover, simple alkyl chains could be introduced between the two functional moieties of 2-methyl-3,4-HOPO-diamine hybrids **IIa-1** and **IIa-2** carrying out a condensation of amino precursors **4** and **5** with maltol benzyl ether respectively. A nucleophilic substitution reaction between new diamine scaffolds **7–8** bearing an iodo group in their side chain and 3,2-HOPO benzyl ether gave the analogs **IIa-3** and **IIa-4**. A new compound **IIb-1** bearing a piperazine-1,4-dialkyl spacer was also obtained by the reduction of the corresponding piperazine-1,4-diamide product derived from the coupling of **6** and **10b**.

Since the synthesis of the diamine building blocks **3**, **4** and **6** as well as HOPO ligands **9a–b**, **10a–b**, 3,2-HOPO and maltol benzyl ethers have been previously described, we focused here only on the preparation of the new precursors **2**, **5**, **7** and **8**.

#### 2.1.1. Synthesis of New Diamine Building Blocks **2**, **5**, **7** and **8**

**Scheme 1:**
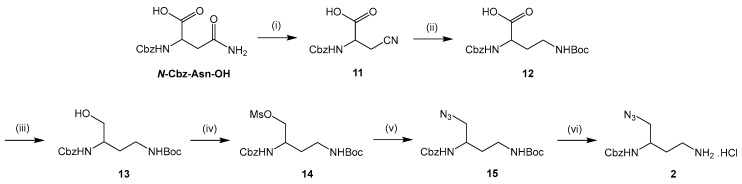
Synthesis of new diamine building block precursor **2**. Reagents and conditions: (**i**) TFAA (1.2 equiv), pyridine (5 equiv), THF, rt, overnight, 92%; (**ii**) NaBH_4_ (7 equiv), NiCl_2_.6H_2_O (0.1 equiv), Boc_2_O (2 equiv), MeOH, 0 °C then rt, 1 h, 96%; (**iii**) 1) ClCOOEt (1 equiv), NMM (1 equiv), THF, −10 °C, 10 min, 2) NaBH_4_ (2.7 equiv), THF/MeOH 1:5, 0 °C, 1 h, 62%; (**iv**) MsCl (1.1 equiv), Et_3_N (1.1 equiv), CH_2_Cl_2_, 0 °C, 1 h, 99%; (**v**) NaN_3_ (2 equiv), DMF, 60 °C, under Ar, 5 h, 79% and (**vi**) 4 N HCl in 1,4-dioxane (30 equiv), 1,4-dioxane, rt, 36 h, 61%.

As shown in Scheme 1 and according to previously described reaction conditions [8], a new azide precursor **2** was prepared starting from commercially available *N*-carboxybenzyl L-asparagine (*N*-Cbz-Asn-OH). The dehydration of its side-chain amide function using trifluoroacetic anhydride in pyridine first provided the cyano intermediate **11** in 92% yield. The corresponding *N*-Boc amino derivative **12** was subsequently obtained after NaBH_4_ reduction of nitrile function in the presence of NiCl_2_.6H_2_O as a catalyst and (Boc)_2_O. The conversion of acid function into primary alcohol followed by mesylate formation and treatment with NaN_3_ afforded the azide intermediate **15** in good yields. Removal of Boc-protecting group was performed to furnish the attempted C2 alkyl side-chain amino ligand **2** as hydrochloride salt.

**Scheme 2:**
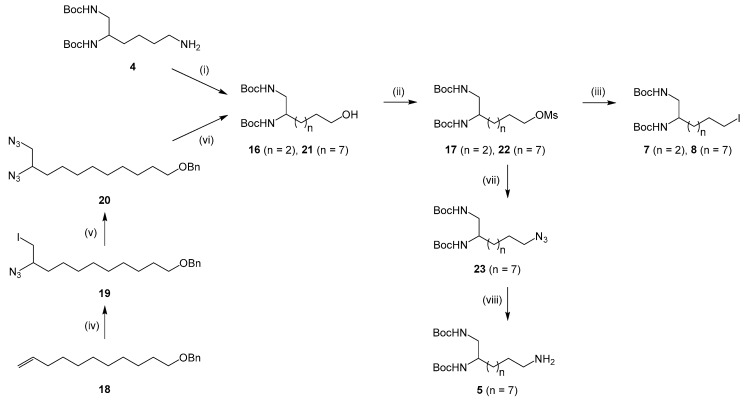
Synthesis of new diamine building blocks **5**, **7** and **8**. Reagents and conditions: (**i**) Na_2_[Fe(CN)_5_NO].2H_2_O (2.5 equiv), 1,4-dioxane/H_2_O 1:1, 65 °C, 16 h, 24%; (**ii**) MsCl (1.1 equiv), Et_3_N (1.1 equiv), CH_2_Cl_2_, 0 °C, 2 h, 80–91%; (**iii**) NaI (5 equiv), acetone, rt, 10–16 h, 53–79%; (**iv**) ICl (1.3 equiv), NaN_3_ (3.3 equiv), MeCN, −15 °C then rt, under Ar, 2 h, 70%; (**v**) NaN_3_ (3 equiv), DMSO, rt, under Ar, 20 h, 78%; (**vi**) 1) H_2_, Pd/C (10% w/w), 1 N aqueous HCl (2 equiv), MeOH, rt, 24 h, 2) Boc_2_O (1.6 equiv), Et_3_N (6.9 equiv), 1,4-dioxane, rt, 2 h, 75%; (**vii**) NaN_3_ (2.5 equiv), DMF, 60 °C, under Ar, overnight, 82% and (**viii**) H_2_, Pd/C (10% w/w), MeOH, rt, 2 h, 88%.

Diamine building blocks **3** and **4** have previously been synthesized by the transformation of respective *α*-carboxylic function of L-ornithine and L-lysine into the amine group [8]. Compound **4** was subjected to the oxidative deamination by sodium nitroprusside to give the corresponding hydroxyl intermediate **16** (Scheme 2) [14]. In addition, the iodo-azide adduct **19** of 10-undecenol benzyl ether **18** was synthesized in 70% yield according to the literature procedure [15]. Iodo group of **19** was then converted into an azide function to provide the compound **20** in 78% yield [16]. This was reduced to the corresponding *N,N’*-di-Boc amine hydroxyl derivative **21** by catalytic hydrogenation in the presence of di-tert-butyl dicarbonate ((Boc)_2_O). Finally, new diamine building blocks **7** and **8** bearing a side-chain iodo group were successfully synthesized after (i) the conversion of the hydroxy group of intermediates **16** and **21** to the corresponding mesylates **17** and **22**, respectively, and (ii) by the iodo substitution of the latter compounds [17]. Furthermore, the transformation of mesylate **22** into the corresponding azide derivative **23** and its subsequent reduction afforded the C9 alkyl side-chain amino analog **5**.

#### 2.1.2. Synthesis of New Multifunctional Scavengers of Series **Ia-b** and **IIa-b** by Coupling of Diamine Building Blocks with HOPO Ligands

A hydroxypyridinone moiety was introduced as a well-known iron chelator pharmacophore to complete the hybridization of our two new series of AGE/ALE inhibitors [18]. Different key coupling steps were designed to build various linkers between the two scavenging cores. Herein, the series **Ia-b** was characterized by an amide or piperazine-1,4-diamide spacer whereas the series **IIa-b** was based on an alkyl or piperazine-1,4-dialkyl linker, respectively.

**Scheme 3:**
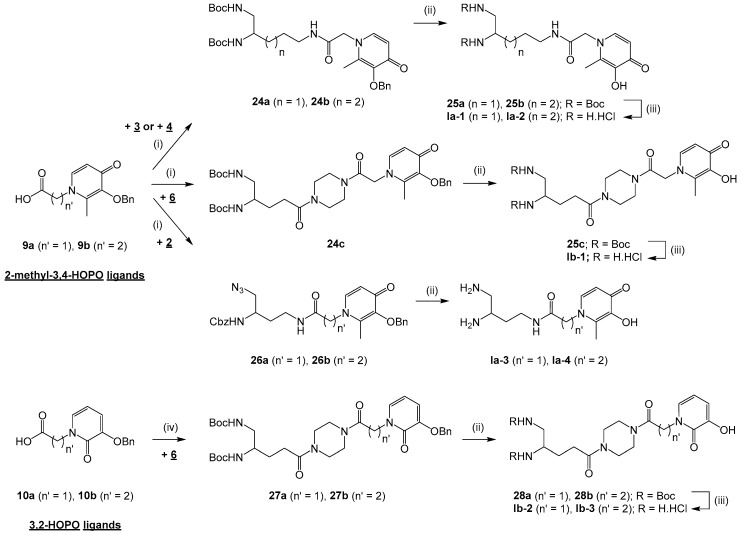
Synthesis of HOPO-diamine hybrids of series **Ia-b**. Reagents and conditions: (**i**) **2**, **3**, **4** or **6** (1 equiv), EDC (1.2–1.4 equiv), HOBt (1–1.2 equiv), Et_3_N (0–1.2 equiv), CH_2_Cl_2_ or THF or THF/DMF 8:1 to 11:1, 0 °C then rt, 6–24 h, 51–70%; (**ii**) H_2_, Pd/C or Pd(OH)_2_/C (10% w/w), MeOH, rt, 6–48 h; 71%–100%; (**iii**) 4 N HCl in 1,4-dioxane (20–30 equiv), 1,4-dioxane, rt, 1.5–36 h, 78–100%; (iv) 1) NHS (1.5 equiv), DCC (1.2 equiv), 1,4-dioxane or CH_2_Cl_2_, rt, overnight, 2) 6 (1.2–1.7 equiv), Et_3_N (3–4 equiv), CH_2_Cl_2_, rt, 48 h, 25–56%.

To develop the series **Ia-b**, a pseudopeptidic coupling of 3,2-HOPO and 2-methyl-3,4-HOPO carboxylic derivatives with various diamine building blocks bearing a side-chain amino group was carried out according to the known procedures (Scheme 3). In particular, HOPO acetic acids **9a** and **10a** allowed the synthesis of new amide or piperazine-1,4-diamide hybrids **24a-c** and **27a** bearing shorter linkers in comparison with our previous analog series represented by compounds **27b**. The synthesis of final compounds **Ia-1**, **Ia-2** and **Ib-1** to **Ib-3** was achieved by the cleavage of HOPO benzyl-protecting group by catalytic hydrogenation followed by *N,N’*-di-Boc removal by treatment with 4N HCl in 1,4-dioxane in excellent yields. Likewise, the pseudopeptidic coupling of diamine building block precursor **2** with 2-methyl-3,4-HOPO ligands **9a-b** provided new hybrid azide intermediates **26a-b**. In this synthetic pathway, a subsequent catalytic hydrogenation simultaneously allowed azide reduction and benzyl and *N*-Cbz removal to directly afford desired final products **Ia-3** and **Ia-4** in 98–100% yield.

**Scheme 4:**
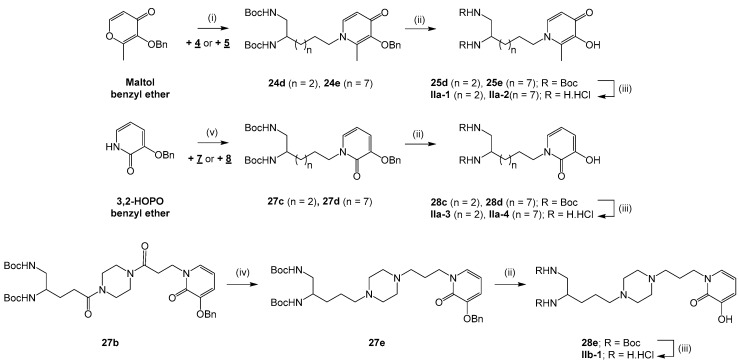
Synthesis of HOPO-diamine hybrids of series **IIa-b**. Reagents and conditions: (**i**) **4** or **5** (0.7–0.9 equiv), NaOH (0.5–2.6 equiv), EtOH/H_2_O 1:1 to 1:2, reflux, 18–24 h, 61–63%; (**ii**) H_2_, Pd/C (10% w/w), MeOH, rt, 3–8 h; 66–91%; (**iii**) 4 N HCl in 1,4-dioxane (20 equiv), 1,4-dioxane, rt, 2–18 h, 60–100%; (**iv**) 2M BH_3_.Me_2_S (5 equiv) in THF, THF, reflux, 2 h, 44%; (**v**) **7** or **8** (1 equiv), NaH 60% dispersion in mineral oil (1 equiv), DMF, 0 °C to rt, under Ar, 2 h, 62–72%.

As shown in Scheme 4, the other new AGE/ALE inhibitors that possess a simple C4 or C9 alkyl or piperazine-1,4-dialkyl linker between the two pharmacophores were successfully synthesized to constitute the series **IIa-b**. The 2-methyl-3,4-HOPO derivatives **24d-e** were prepared carrying out a condensation of maltol benzyl ether with amino starting blocks **4** and **5** following the protocol of Santos et al. [19]. Furthermore, a nucleophilic substitution of the iodo group of diamine derivatives **7** and **8** by 3,2-HOPO benzyl ether was achieved using sodium hydride in DMF to give hybrid products **27c-d** in 72 and 62% yield, respectively [13]. A novel piperazine-1,4-dialkyl intermediate **27e** was also prepared by reduction of the piperazine-1,4-diamide spacer of the corresponding analog **27b** using borane-methyl sulfide complex following the protocol of Boschelli et al. [20]. The attempted compounds **IIa-1** to **IIa-4** and **IIb-1** were finally provided via an *O*-debenzylation and diamine deprotection sequence as a promising novel series of hydroxypyridinone-diamine hybrids. 

Various new final products have been synthesized in 7–11 steps and constitute two novel series of HOPO-diamine hybrids: i) the series **I** was obtained in 13–39% overall yields and can be arranged in **Ia** with compounds **Ia-1** to **Ia-4** bearing an amide linker and **Ib** with compounds **Ib-1** to **Ib-3** bearing a piperazine-1,4-diamide linker and ii) the series **II** was prepared in 2–57% overall yields and can be arranged in **IIa** with compounds **IIa-1** to **IIa-4** possessing an alkyl spacer and **IIb** with compound **IIb-1** possessing a piperazine-1,4-dialkyl spacer. The impact of these different pharmacomodulations on their ADME, physicochemical and biological properties as multifunctional AGE/ALE inhibitors, with respect to lead compound **1**, in particular, have then been investigated.

### 2.2. QikProp-Predicted ADME Properties 

**Figure 2:**
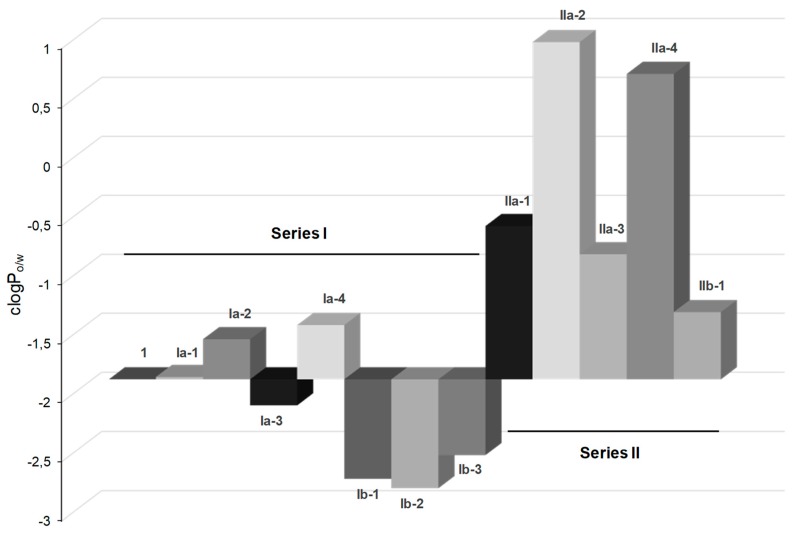
Calculated logP_o/w_ repartition between the different HOPO-diamine hybrid series.

A prediction of the ADME properties of newly synthesized products was performed using QikProp, a Schrödinger software. As shown in Figure 2, logP_o/w_ (octanol/water partition coefficient) values calculated for derivatives of series **IIa** appeared much superior to that of lead compound **1** (Table 2: clogP_o/w_ of −1.88). Indeed, a rise of one to three logarithmic units was found for clogP_o/w_ of novel HOPO-alkyl-diamine hybrids **IIa-1** to **IIa-4**. There was no significant difference in terms of clogP_o/w_ between 3,2-HOPO derivatives **IIa-3** and **IIa-4** and their 2-methyl-3,4-HOPO analogs **IIa-1** and **IIa-2**, respectively. However, the lipophilicity strongly increased in this series with the length of the alkyl chain between the diamine and HOPO pharmacophores (Table 2: **IIa-4** vs. **IIa-3** and **IIa-2** vs. **IIa-1** bearing a C9 vs. C4 alkyl linker, with clogP_o/w_ of 0.71 vs. −0.82 and 0.98 vs. −0.58, respectively). In comparison with the series **IIa**, the amide derivatives of series **Ia** still presented a high predicted hydrophilicity (Figure 2: **Ia**-1 to **Ia-4** vs. **IIa-1** and **IIa-2**). The increase in the size of the amide linker slightly affected the clogP_o/w_ values (Table 2: **Ia**-3 vs. **Ia-2** with clogP_o/w_ of −2.10 vs. −1.54, respectively), but they remained in the range of that of lead compound **1**. In the series **I**, the introduction of a piperazine cycle inside the spacer greatly decreased the clogP_o/w_ for 3,2-HOPO derivatives whereas the nature of HOPO moiety seemed to have no particular impact (Figure 2: **Ib-2** vs. **1** and **Ib-1**). However, the reduction of piperazine-1,4-diamide spacer of **Ib-3** into a piperazine-1,4-dialkyl spacer led to an increase of more than one logarithmic unit for clogP_o/w_ value of **IIb-1** (Table 2: clogP_o/w_ of −2.52 and −1.31, respectively). As expected, the nature and the length of the spacer significantly affected the lipophilicity of HOPO-diamine hybrids. In particular, the introduction of an alkyl linker in the series **IIa** greatly improved it. This was correlated with an important increase of logBB (brain/blood partition coefficient) value calculated for the product **IIa-3** compared to that of lead compound **1** (Table 2: clogBB of −0.86 and −1.77, respectively). In addition, enhanced apparent Caco-2 cell (a gutblood barrier model) and MDCK cell (a blood–brain barrier model) permeabilities to this new alkyl derivative were found (Table 2: **IIa-3** vs. **1** with P_Caco_ of 10.21 vs. 1.73 and P_MDCK_ of 4.27 vs. 1.07 nm/sec, respectively). It is also particularly interesting to note that this product exhibited the most important central nervous system activity on a −2 (inactive) to +2 (active) QikProp-established scale (Table 2: **IIa-3** vs. **1** with CNS activity of −1 vs. −2). Finally, in view of these predicted ADME properties and especially its improved capacity to cross the blood brain barrier, the HOPO-alkyl-diamine hybrid **IIa-3** appears as a novel promising drug candidate for treating AD. Now, a further screening is to be carried out to confirm its good RCS trapping, antioxidant and Cu^2+^-chelating capacities.

### 2.3. Physicochemical Evaluations: Oxygen Radical Absorbance Capacity (ORAC), RCS Trapping and Cu^2+^-Chelating Assays

**Figure 3:**
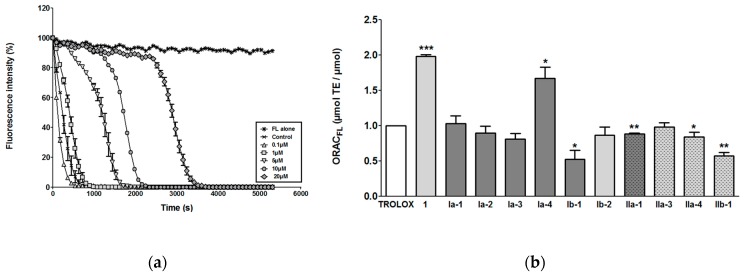
(**a**) Fluorescein (FL) fluorescence decay curve induced by AAPH. Tested compounds (0.1–20 µM) or trolox standard (1–50 µM) were pre-incubated with FL (12 nM) in D-PBS (pH 7.4) at 37 °C for at least 30 min. The addition of AAPH reagent (30 mM) led to FL disappearance and the remaining FL fluorescence (λ_Ex_: 485 nm; λ_Em_: 520 nm) was measured every 90 s for 60 cycles. Represented data show results obtained in the absence (control) or the presence of compound **IIa-3** at different concentrations and are expressed as means ± SEM of triplicates that form a representative experiment among the at least performed three independent ones and (**b**) oxygen radical absorbance capacity assay using fluorescein (ORAC_FL_) values of new HOPO-diamine hybrids, lead compound **1** and references. Bars represent the means ± SEM calculated at 10 µM of at least three independent experiments performed in triplicate. * *p* < 0.05; ** *p* < 0.01; *** *p* < 0.001 vs. Trolox (Student’s *t* test: *p* values <0.05 were considered significant).

In order to evaluate the antioxidant activity of new synthesized HOPO-diamine hybrids, an oxygen radical absorbance capacity assay using fluorescein (ORAC_FL_) was performed [21,22]. The fluorescein (FL) fluorescence decay induced by AAPH reagent, a peroxyl radical generator was followed in time at 37 °C. The measurement of the area under the curve (AUC) of the sample in comparison with the control corresponding to an absence of antioxidant allowed us to determine the protective effect of tested compounds (Figure 3a). Trolox, a water-soluble vitamin E analog was used as a standard for the calculation of ORAC_FL_ values at 10 µM expressed as µmol trolox equivalent (TE)/µmol of tested compound with respect to the linear equation of its calibration curve (Net AUC vs. concentration) [23]. As shown in Figure 3b, the majority of newly designed products appeared to be efficient ROS scavengers (ORAC_FL_ values ≥0.8 µmol TE/µmol, except for **IIb-1** and **Ib-1**), especially compared to the very weak protective effect of carnosine, reported as a good antioxidant (Table 3: ORAC_FL_ value of 0.08 µmol TE/µmol) [24]. The HOPO moiety previously revealed to be responsible for the radical trapping activity [8]. Nevertheless, the lead compound **1** remains the most potent HOPO-diamine hybrid (Table 3: ORAC_FL_ value of 1.96 µmol TE/µmol). In the two novel series, the 3,2-HOPO derivatives exhibited slightly better antioxidant properties than their 2-methyl-3,4-HOPO analogs (Figure 3b: **Ib-2** vs. **Ib-1** and **IIa-3** vs. **IIa-1**). Furthermore, some pharmacomodulations carried out on the linker could affect this protective effect. In the series **I**, the compound **Ia-4** turned out to be almost as active as lead compound **1** whereas its analog **Ia-3** bearing a one-carbon shorter amide linker provided a less interesting result (Table 3: ORAC_FL_ values of 1.67 and 0.81 µmol TE/µmol, respectively). The simple removal of the carbonyl group from HOPO moiety greatly improved its reactivity. However, the introduction of a piperazine cycle inside the spacer significantly decreased ROS scavenging capacities (Figure 3b: **1** vs. **Ib-2** and **Ia-2** vs. **Ib-1**). In the series **II**, there was a negative impact of the reduction of piperazine-1,4-diamide linker into piperazine-1,4-dialkyl spacer (Table 3: ORAC_FL_ values of 0.99 and 0.57 µmol TE/µmol for **Ib-3** and **IIb-1**, respectively). Finally, the 3,2-HOPO derivative **IIa-3** possessing a C4 alkyl spacer appeared slightly more efficient than its analog **IIa-4** bearing a C9 alkyl linker (Table 3: ORAC_FL_ values of 0.98 and 0.84 µmol TE/µmol, respectively). Judging from its good conserved antioxidant activity and with its promising Qikprop-predicted ADME properties in mind, this HOPO-alkyl-diamine hybrid could be considered as a new lead compound. Its RCS trapping and Cu^2+^-chelating capacities have been evaluated according to previously described conditions [8].

**Figure 4:**
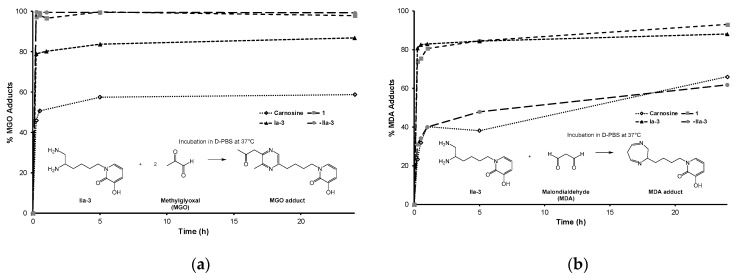
Kinetic curves representing the formation of (**a**) MGO or (**b**) MDA adducts in the presence of different scavengers. Tested compounds (10 mM) dissolved in D-PBS (pH 7.4) were incubated with MGO or MDA (20 mM) at 37 °C for 24 h. Samples collected at regular time intervals were subsequently analyzed by LCMS. Area under the curve (AUC) of the MGO or MDA adduct peak and remaining free scavenger peak were measured on UV chromatogram at 190 nm. Data are expressed as percentage of total MGO or MDA adducts compared with remaining free scavenger for a representative single sample. ND: Not determined.

A kinetic study of RCS adduct formation was assessed by LCMS after the incubation of new key diamine compounds **IIa-3** and **Ia-3** with MGO or MDA at 37 °C for 24 h (Figure 4). Indeed, the appearance of already described major pyrazine or 1,4-diazepine adducts could be followed on UV chromatogram at 190 nm, respectively [8]. AUC of the total peak of adducts was compared with the remaining free scavenger peak for samples collected at regular time intervals. As shown in Table 3, the reactivity of primary vicinal diamine function of **IIa-3** and **Ia-3** with dicarbonyl compounds remains as interesting as that of lead compound **1**. Newly designed derivatives were found to be very potent MGO scavengers. In particular, 100% of MGO adduct formation was detected in 15 min of incubation with **IIa-3** vs. only 46% with carnosine used as reference [24]. However, the MDA trapping capacity of this HOPO-alkyl-diamine hybrid revealed weaker than that of **Ia-3** and lead compound **1** both bearing an amide linker (Table 3: 40% vs. 83% and 81% MDA adduct formation, respectively, in 1 h of incubation). The modification of the spatial geometry of the hybrid structure induced by the introduction of an alkyl spacer seems to lead to a lower reactivity of diamine function with bulky RCS like α,β-unsaturated aldehydes.

**Figure 5:**
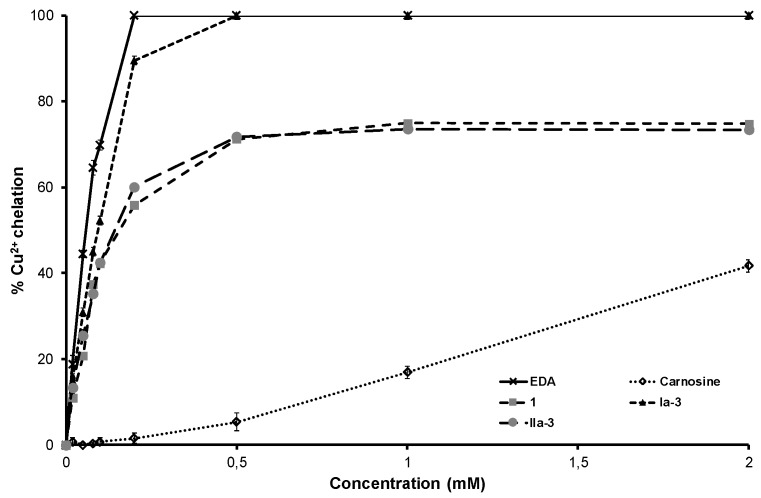
Evaluation of Cu^2+^-chelating capacity of new HOPO-diamine hybrids and references. Tested compounds (0–2 mM) were incubated with CuSO_4._5H_2_O (120 µM) in a 10 mM hexamine/HCl buffer containing 10 mM KCl (pH 5.0) for 10 min at rt. Murexide (50 µM), used as a complexometric indicator reacting with remaining free Cu^2+^, was added and the mixture was incubated for additional 1 min at rt. The absorbance ratio A_485_/A_520_ (λ_max_ of Cu^2+^/murexide complex: 485 nm and λ_max_ of free murexide: 520 nm) provided remaining free Cu^2+^ concentration with respect to calibration curves (A_485_/A_520_ vs. Cu^2+^ concentration). Knowing the total quantity of metal ions introduced in the reaction mixture (control conditions without tested product), percentage of Cu^2+^ chelation by tested compounds was calculated by difference. Data are presented as means ± SEM of triplicates.

The evaluation of Cu^2+^-chelating properties of derivatives **IIa-3** and **Ia-3** representing the two new series of AGE/ALE inhibitors was performed using murexide as complexometric indicator. Tested compounds at different concentrations were incubated with CuSO_4._5H_2_O for 10 min at rt. Murexide was subsequently introduced into the mixture and reacted with remaining free Cu^2+^ for additional 1 min. The absorbance ratio A_485_/A_520_ (λ_max_ of Cu^2+^/murexide complex: 485 nm and λ_max_ of free murexide: 520 nm) furnished the remaining free Cu^2+^ concentration with respect to calibration curves (A_485_/A_520_ vs. Cu^2+^ concentration). Knowing the total quantity of metal ions added into the reaction mixture (control conditions without tested product), proportion of Cu^2+^ chelation by HOPO-diamine hybrids was estimated by difference. Newly synthesized compounds **IIa-3** and **Ia-3** appeared to be much more potent than carnosine in complexing Cu^2+^ (Figure 5). In the hybrid structure, it has been previously demonstrated that the role of HOPO moiety is more essential than that of the diamine function as far as the Cu^2+^-chelating activity is concerned [8]. As shown in Table 3, the 2-methyl-3,4-HOPO derivative **Ia-3** showed 83% Cu^2+^ chelation at 200 µM and revealed almost as efficient as the positive standard EDA (ethylenediamine) whereas the 2,3-HOPO one **IIa-3** exhibited its chelating capacity in only 60%. This provides novel evidence that demonstrates the slightly weaker biometal complexing properties of the 2,3-HOPO moiety vs. the 2-methyl-3,4-HOPO one. However, judging from similar interesting results obtained with **IIa-3** and lead compound **1**, the nature of the linker seems to have no particular impact in Cu^2+^ chelation (Figure 5).

Finally, in view of its particularly promising predicted ADME properties and its good conserved RCS, ROS and biometal scavenging efficiency, the newly designed 3,2-HOPO-alkyl-diamine **IIa-3** can be selected as a novel lead compound.

### 2.4. Biological Evaluations

#### 2.4.1. Cell Viability

**Figure 6:**
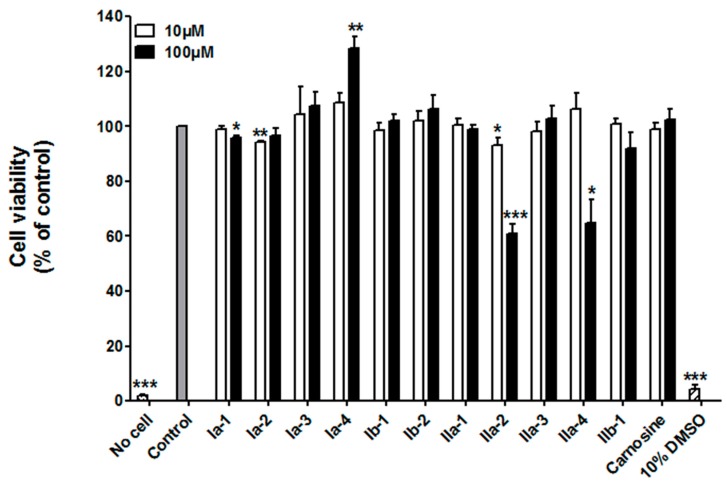
Evaluation of cell viability. PC12 cells were incubated with 10 µM or 100 µM of the different multifunctional diamine derivatives for 48 h. DMSO was used as a positive cytotoxicity control. Results are expressed as % of control (non-treated cells). Bars represent the mean ± SEM of at least three independent experiments performed in triplicate. * *p* < 0.05; ** *p* < 0.01; *** *p* < 0.001 vs. control (Student’s t test: *p* values <0.05 were considered significant).

Cytotoxicity of the novel HOPO-diamine hybrids was evaluated on an *in vitro* neuronal-like cell model, the rat pheochromocytoma cell-line PC12 widely used to study neurodegenerative diseases [25,26]. A sensitive colorimetric CCK-8 (cell counting kit-8) assay was performed to determine cell viability [27]. In the cell, mitochondrial dehydrogenases reduced the WST-8 reagent in the presence of 1-methoxy PMS used as an electron carrier to give a water-soluble formazan dye. The number of living cells was estimated by medium absorbance measurement after incubation with different concentrations of tested compounds for 48 h. A positive cytotoxicity standard was assessed by adding 10% DMSO and cell viability was expressed as % of control conditions (non-treated cells). Except for compounds **IIa-2** and **IIa-4** bearing a C9 alkyl linker and to a lesser extent for compounds **Ia-1** and **Ia-2**, no cytotoxicity of new multifunctional diamine derivatives was detected in PC12 cells after 48 h of treatment at 10 µM as well as at 100 µM (Figure 6). The increased cellular toxicity of hybrids **IIa-2** and **IIa-4** could be explained by the well-known ability of flexible long alkyl chains to induce membrane disruption and cytolysis slotting into the phospholipid bilayer [28].

#### 2.4.2. *In Vitro* MGO-Induced Apoptosis Inhibition in the Model AD Cell-Line PC12 

**Figure 7:**
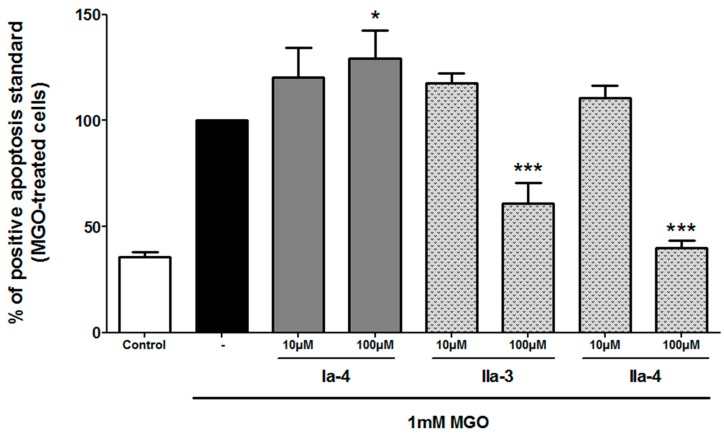
*In vitro* evaluation of MGO-induced apoptosis inhibition in neuronal-like PC12 cells. PC12 cells were incubated for 24 h in media (control), and with MGO (1 mM) in the absence or the presence of either 10 µM or 100 µM of key AGE/ALE inhibitors Ia-4, IIa-3 and IIa-4. At the end of the incubation, the cells were lysed and analyzed for DNA fragmentation. Bars represent the means ± SEM of at least three independent experiments performed in triplicate. * *p* < 0.05; *** *p* < 0.001 vs. control (Student’s *t* test: *p* values <0.05 were considered significant).

With the aim of assessing their ability to limit MGO-induced apoptosis in AD, a pre-treatment of PC12 cells with compounds **Ia-4**, **IIa-3** and **IIa-4** representing the two new series of AGE/ALE inhibitors were carried out. After incubation in the presence of MGO at 37 °C, apoptosis level was evaluated by ELISA detection of DNA fragmentation and reflected by a measurement of optical density (OD) [29,30]. Cells incubated without MGO represented control conditions and a positive apoptosis standard was constituted in the presence of MGO, but in the absence of the tested product. As shown in Figure 7, the apoptosis level was greatly decreased in the presence of HOPO-alkyl-diamine derivatives **IIa-3** and **IIa-4** at 100 µM whereas there was no significant effect of the compound **Ia-4** bearing an amide linker neither at 10 µM nor at 100 µM. Furthermore, the new multifunctional scavengers **IIa-3** and **IIa-4** revealed to be more efficient than lead compound 1 to inhibit MGO-induced apoptosis in PC12 cells (21% of apoptosis inhibition with 1 at 100 µM (previously reported result [8]) vs. 39% and 60% with **IIa-3** and **IIa-4**, respectively). Considering their similar trapping abilities, these results can be explained by the increased lipophilicity of **IIa-3** and **IIa-4** associated with the enhanced QikProp-predicted cellular permeability to these compounds (Table 2). However, the long-chain alkyl hybrid **IIa-4** must be ruled out for further investigations because of its detected cytotoxicity in PC12 cells. Finally, together with its RCS, ROS and biometal scavenging efficiency and its good QikProp-predicted ADME properties, HOPO-alkyl-diamine derivative **IIa-3** exhibited an *in vitro* potent biological activity. These new data constitute a promising evidence of its improved druglikeness and capacity to prevent biometal-mediated oxidative and carbonyl stress progression in the PC12 cell-line model of AD.

## 3. Materials and Methods

### 3.1. Generalities

All reagents and solvents were obtained from commercial suppliers and were used without further purification. Anhydrous solvents were dried with the PureSolv^®^ MD 5 solvent purification system. Column chromatography was performed over Merck Silica gel 60 Å (40–63 μm) or using a Grace Davison Purification Reveleris^®^ Flash system. Routine monitoring of reactions was carried out using Merck thin layer chromatography (TLC) Silica gel 60 F254 plates, visualized under UV light (254 nm) and revealed using a phosphomolybdic acid 95% solution in ethanol. Melting points (mp) were determined on a Stuart SMP3 device. IR measurements were performed on a Jasco FT/IR-4200 system fitted with an ATR-golden gate and were reported using the frequency of absorption (cm^−1^). nuclear magnetic resonance (NMR) spectra were recorded on a Bruker 400 (or 300) cryosonde NMR instrument. Chemical shifts (*δ*) are expressed in parts per million (ppm) downfield from tetramethylsilane as an internal standard and were referenced to the deuterated solvent. ^1^H, ^13^C and Q-DEPT data were reported in the order of chemical shift, multiplicity (s = singlet, br s = broad singlet, d = doublet, t = triplet, q = quartet, quint = quintet and m = multiplet), integration, coupling constants in Hertz (Hz). Negative peaks (np) are quoted for Q-DEPT NMR spectra. High-resolution mass spectra (HRMS) were obtained from a Micromass-Waters Q-TOF Ultima spectrometer, in electrospray ionization (ESI) mode (positive or negative). For liquid chromatography coupled with mass spectrometry (LCMS), UV chromatograms and mass spectra were obtained from a Shimadzu LCMS-2020 system, at 190 nm and by positive ESI-MS interface (detection mode: scan, interface voltage: tuning file, DL voltage: 100 V, Q-array DC: 40 V, Q-array RF: 40 V). The gradient elution was performed on a Phenomenex Kinetex^®^ HPLC C18 column using an injection volume of 1–2 µL and a mobile phase composed of water/acetonitrile (solvent A/solvent B) with 0.1% formic acid (98:2 during 2 min, 55:45 during 2 min and 45:55 during 3 min with a flow of 0.3 mL/min at 40 °C).

### 3.2. Chemical Synthesis

#### 3.2.1. Synthesis of New Diamine Building Block **2**

2-(((Benzyloxy)carbonyl)amino)-3-cyanopropanoic acid (**11**). To a solution of *N*-Cbz-Asn-OH (10 mmol) in THF (10 mL) cooled at −10 °C were added trifluoroacetic anhydride (1.2 equiv) and pyridine (5 equiv). The mixture was stirred at rt for 18 h. After evaporating THF under reduced pressure, a 1 N NaOH solution (20 mL) was added and pyridine was extracted with Et_2_O (2 × 20 mL). The aqueous layer was acidified to pH 2 with a 6 N HCl solution and extracted with EtOAc (2 × 20 mL). The organic phase was washed with brine (2 × 20 mL), dried over Na_2_SO_4_ and concentrated in vacuo to give compound **11** as a white solid (2.3 g, 92%). mp 145 °C. *ν*_max_: 3410, 3331, 3220, 1693, 1533, 1262, 1060, 735, 606 cm^−1^. ^1^H NMR (400 MHz, methanol-*d_4_*): *δ* 7.38–7.29 (m, 5H), 5.13 (s, 2H), 4.97 (br s, 1H), 4.51 (t, *^3^J* = 6.7 Hz, 1H), 3.08–2.91 (m, 2H). Q-DEPT NMR (101 MHz, methanol-*d_4_*): *δ* 171.8 (np), 137.9 (np), 129.4 (2C), 128.9, 128.6 (2C), 118.2 (np), 67.7 (np), 51.6, 21.1 (np). HRMS (ESI): *m/z* calculated for C_12_H_12_N_2_O_4_Na, [M + Na]^+^ 271.0695, found 271.0687.

2-(((Benzyloxy)carbonyl)amino)-4-((tert-butoxycarbonyl)amino)butanoic acid (**12**). To a solution of compound **11** (8 mmol) in dry methanol (60 mL) cooled at 0 °C were added Boc_2_O (2 equiv) and NiCl_2_.6H_2_O (0.1 equiv). NaBH_4_ (7 equiv) was then introduced in several portions over a period of 45 min. The mixture was stirred at rt for 1 h and diethylenetriamine (1 equiv) was added to quench NiCl_2_.6H_2_O. After stirring further for 1 h, methanol was evaporated and the resulting product was extracted with Et_2_O (2 × 80 mL). The organic phase was washed with a saturated NaHCO_3_ solution (2 × 80 mL) and brine (2 × 80 mL), dried over Na_2_SO_4_ and concentrated in vacuo to give compound **12** as a yellow oil (2.7 g, 96%). *ν*_max_: 3314, 2975, 1689, 1515, 1247, 1162, 1046, 741, 623 cm^−1^. ^1^H NMR (400 MHz, chloroform-*d*): *δ* 8.49 (br s, 1H), 7.34–7.31 (m, 5H), 5.85 (br s, 1H), 5.2 (br s, 1H), 5.09 (s, 2H), 4.40 (m, 1H), 3.38–3.05 (m, 2H), 2.27–2.02 (m, 2H), 1.44 (s, 9H). ^13^C NMR (101 MHz, chloroform-*d*): *δ* 175.0, 158.1, 156.8, 136.3, 128.7 (2C), 128.3 (2C), 128.2, 80.1, 67.2, 51.6, 45.0, 36.3, 28.4 (3C). HRMS (ESI): *m/z* calculated for C_17_H_24_N_2_O_6_Na, [M + Na]^+^ 375.1532, found 375.1519.

Benzyl tert-butyl (4-hydroxybutane-1,3-diyl)dicarbamate (**13**). To a solution of compound **12** (7.4 mmol) in dry THF (15 mL) cooled at −10 °C were added *N*-methylmorpholine (1 equiv) and ethyl chloroformate (1 equiv). After stirring for 10 min, NaBH_4_ (2.7 equiv) was introduced in one portion. The mixture was diluted in methanol (75 mL) and further stirred at 0 °C for 1 h. THF and methanol were evaporated and the resulting product was extracted with EtOAc (2 × 100 mL). The organic phase was washed with a saturated NaHCO_3_ solution (2 × 100 mL) and brine (2 × 100 mL), dried over Na_2_SO_4_ and concentrated in vacuo to give compound **13** as a white pasty solid (1.6 g, 62%) after purification by column chromatography on silica gel (EtOAc/Cyclohexane 60:40). *ν*_max_: 3343, 2939, 1679, 1526, 1247, 1166, 1061, 628 cm^−1^. ^1^H NMR (400 MHz, chloroform-*d*): *δ* 7.29–7.27 (m, 5H), 5.38 (br s, 1H), 5.19 (br s, 1H), 5.10 (s, 2H), 3.65–3.49 (m, 3H), 3.23 (br s, 1H), 3.08–3.04 (m, 1H), 2.94–2.86 (m, 1H), 1.67–1.50 (m, 2H), 1.35 (s, 9H). ^13^C NMR (101 MHz, chloroform-*d*): *δ* 156.9, 156.3, 136.4, 128.6 (2C), 128.2, 128.0 (2C), 79.4, 66.9, 64.9, 50.5, 37.2, 32.0, 28.4 (3C). HRMS (ESI): *m/z* calculated for C_17_H_26_N_2_O_5_Na, [M + Na]^+^ 361.1739, found 361.1747.

2-(((Benzyloxy)carbonyl)amino)-4-((tert-butoxycarbonyl)amino)butyl methanesulfonate (**14**). Compound **13** (4.4 mmol) was dissolved in CH_2_Cl_2_ (40 mL) and the solution was cooled at 0 °C. Triethylamine (1.1 equiv) and mesyl chloride (1.1 equiv) were then added. The mixture was stirred at 0 °C for 1 h and the resulting product was extracted with CH_2_Cl_2_ (2 × 60 mL). The organic phase was washed with a 0,5 N HCl solution (2 × 60 mL), a 5% NaHCO_3_ solution (2 × 60 mL) and brine (2 × 60 mL), dried over Na_2_SO_4_ and concentrated in vacuo to give compound **14** as a brown oil (1.8 g, 99%). *ν*_max_: 3250, 3059, 2947, 1726, 1154, 1037, 938, 772 cm^−1^. ^1^H NMR (400 MHz, chloroform-*d*): *δ* 7.31–7.26 (m, 5H), 5.20 (br s, 1H), 5.03 (s, 2H), 4.93 (br s, 1H), 4.14–4.20 (m, 2H), 3.93–3.89 (m, 1H), 3.31–3.27 (m, 1H), 3.07–3.03 (m, 1H), 2.95 (s, 3H), 1.69–1.55 (m, 2H), 1.36 (s, 9H). ^13^C NMR (101 MHz, chloroform-*d*): *δ* 156.3, 156.1, 136.2, 128.6 (2C), 128.3, 128.1 (2C), 79.5, 71.0, 67.0, 48.0, 37.3, 34.5, 31.8, 28.4 (3C). HRMS (ESI): *m/z* calculated for C_18_H_28_N_2_O_7_SNa, [M + Na]^+^ 439.1515, found 439.1523.

Benzyl tert-butyl (4-azidobutane-1,3-diyl)dicarbamate (**15**). Compound **14** (3.8 mmol) was dissolved in DMF (5 mL). Sodium azide (2 equiv) was then added and the mixture was heated at 60 °C for 5 h under argon. The solvent was removed and the residue was taken up in EtOAc (3 × 25 mL). The organic phase was washed with brine (3 × 25 mL), dried over Na_2_SO_4_ and concentrated in vacuo. The crude material was purified by column chromatography on silica gel (EtOAc/Cyclohexane 50:50) to give compound **15** as a white pasty solid (1.1 g, 79%). *ν*_max_: 3346, 2096, 1680, 1522, 1251, 1162, 1067, 696, 626 cm^−1^. ^1^H NMR (400 MHz, chloroform-*d*): *δ* 7.39–7.33 (m, 5H), 5.20 (br s, 1H), 5.12 (s, 2H), 5.05 (br s, 1H), 3.89–3.86 (m, 1H), 3.49–3.36 (m, 3H), 3.02–2.95 (m, 1H), 1.73–1.56 (m, 2H), 1.45 (s, 9H). Q-DEPT NMR (101 MHz, chloroform-*d*): *δ* 156.3 (np), 156.1 (np), 136.3 (np), 128.6 (2C), 128.2, 128.1 (2C), 79.4 (np), 67.1 (np), 54.8 (np), 48.5, 37.0 (np), 32.8 (np), 28.4 (3C). HRMS (ESI): *m/z* calculated for C_17_H_25_N_5_O_4_Na, [M + Na]^+^ 386.1804, found 386.1814.

Benzyl (4-amino-1-azidobutan-2-yl)carbamate hydrochloride (**2**). To a solution of compound **15** (0.1 mmol) dissolved in 1,4-dioxane (3 mL) was added 4 N HCl solution in 1,4-dioxane (30 equiv). The mixture was stirred at rt for 36 h and concentrated under reduced pressure. The residue was washed with Et_2_O (2 × 3 mL) that was then evaporated *in vacuo* to give compound **2** as a white pasty solid (18 mg, 61%). *ν*_max_: 3298, 3031, 2939, 2893, 2098, 1692, 1525, 1251, 1048, 698 cm^−1^. ^1^H NMR (400 MHz, methanol-*d_4_*): *δ* 7.41–7.29 (m, 5H), 5.23 (s, 2H), 3.83–3.79 (m, 1H), 3.60 (br s, 1H), 3.42 (d, *^3^J* = 5.8 Hz, 2H), 3.01–2.94 (m, 2H), 1.93–1.74 (m, 2H). Q-DEPT NMR (101 MHz, methanol-*d_4_*): *δ* 158.7 (np), 138.0 (np), 129.5 (2C), 129.0, 128.7 (2C), 67.7 (np), 55.3 (np), 50.0, 37.8 (np), 31.2 (np). HRMS (ESI): *m/z* calculated for C_12_H_18_N_5_O_2_, [M + H]^+^ 264.1461, found 264.1453.

#### 3.2.2. Synthesis of New Diamine Building Blocks **5**, **7** and **8**

For compound **18**, synthetic procedure and NMR spectra were in full accordance with those reported in the literature [31]. 

##### Synthesis of Intermediates **17** and **22**

(((10-azido-11-iodoundecyl)oxy)methyl)benzene (**19**). A solution of iodine chloride (1.3 equiv.) in acetonitrile (10 mL) was added dropwise over 10 min to a stirred suspension of sodium azide (3.3 equiv.) in acetonitrile (10 mL) at −15 °C under argon. The compound **18** (10 mmol) was then added at −15 °C over a period of 20 min and the mixture was warmed to rt. Stirring was continued for 2 h and the mixture was worked up by addition of water (20 mL) and extraction with cyclohexane (2 × 40 mL). The organic layer was washed with a 10% aqueous sodium thiosulfate (2 × 40 mL) and brine (2 × 40 mL), dried over Na_2_SO_4_ and concentrated in vacuo. The crude material was purified by column chromatography on silica gel (EtOAc/Cyclohexane 5:95) to give compound **19** as a yellow oil (3 g, 70%). *ν*_max_: 2924, 2852, 2099, 1456, 1272, 1101, 736, 698, 616 cm^−1^. ^1^H NMR (400 MHz, chloroform-*d*): *δ* 7.35–7.30 (m, 4H), 7.30–7.26 (m, 1H), 4.51 (s, 2H), 3.47 (t, *^3^J* = 8.0 Hz, 2H), 3.41–3.36 (m, 1H), 3.30–3.22 (m, 2H), 1.69–1.56 (m, 8H), 1.38–1.36 (m, 6H), 1.27–1.25 (m, 2H). ^13^C NMR (101 MHz, chloroform-*d*): *δ* 139.0, 128.6 (2C), 127.9 (2C), 127.7, 73.1, 70.8, 63.0, 34.7, 30.0 (2C), 29.7, 29.6, 29.4, 26.4, 26.1, 8.8. HRMS (ESI): *m/z* calculated for C_18_H_28_N_3_ONaI, [M + Na]^+^ 452.1175, found 452.1174.

(((10,11-diazidoundecyl)oxy)methyl)benzene (**20**). Compound **19** (6 mmol) was dissolved in DMSO (7 mL) and sodium azide (3 equiv) was added. The mixture was stirred at rt for 20 h under argon and then diluted in EtOAc (3 × 25 mL). The organic phase was washed with brine (3 × 25 mL), dried over Na_2_SO_4_ and concentrated in vacuo. The crude material was purified by column chromatography on silica gel (EtOAc/Cyclohexane 5:95) to give compound **20** as colorless oil (1.6 g, 78%). *ν*_max_: 2931, 2856, 2326, 2103, 1717, 1277, 890, 796, 715, 631 cm^−1^. ^1^H NMR (400 MHz, chloroform-*d*): *δ* 7.35–7.30 (m, 4H), 7.30–7.27 (m, 1H), 4.51 (s, 2H), 3.47 (t, *^3^J* = 8.0 Hz, 2H), 3.44–3.28 (m, 3H), 1.65–1.58 (m, 3H), 1.57–1.51 (m, 3H), 1.45–1.43 (m, 2H), 1.38–1.35 (m, 8H). ^13^C NMR (101 MHz, chloroform-*d*): *δ* 139.0, 128.6 (2C), 127.9 (2C), 127.7, 73.1, 70.7, 62.3, 55.1, 32.0, 30.0, 29.7 (2C), 29.6, 29.5, 26.4, 26.1. HRMS (ESI): *m/z* calculated for C_18_H_28_N_6_ONa, [M + Na]^+^ 367.2222, found 367.2223.

di-tert-butyl (6-hydroxyhexane-1,2-diyl)dicarbamate (**16**). Compound **4** (2 mmol) was dissolved in 1,4-dioxane/H_2_O 1:1 (40 mL) and the mixture was heated to 65 °C. Sodium nitroprusside (2.5 equiv) was added portionwise over a period of 30 min and the reaction mixture was heated for 16 h. After cooling to rt, 1,4-dioxane was removed and the resulting product was extracted with EtOAc (3 × 80 mL). The organic phase was washed with brine (3 × 80 mL), dried over Na_2_SO_4_ and concentrated in vacuo. The crude material was purified by column chromatography on silica gel (EtOAc) to give compound **16** as a white solid (160 mg, 24%). mp 55 °C. *ν*_max_: 3363, 2919, 2852, 1682, 1523, 1448, 1367, 1247, 1160, 1071, 1039, 986, 884, 641 cm^−1^. ^1^H NMR (400 MHz, chloroform-*d*): *δ* 4.99 (br s, 1H), 4.77 (br s, 1H), 3.61–3.57 (m, 3H), 3.16–3.12 (m, 2H), 2.29 (br s, 1H), 1.60–1.51 (m, 2H), 1.49–1.44 (m, 4H), 1.40 (s, 18H). ^13^C NMR (101 MHz, chloroform-*d*): *δ* 156.9, 156.5, 79.6 (2C), 62.4, 51.6, 44.8, 32.7, 32.6, 28.6 (6C), 22.2. HRMS (ESI): *m/z* calculated for C_16_H_32_N_2_O_5_Na, [M + Na]^+^ 355.2209, found 355.2216.

di-tert-butyl (11-hydroxyundecane-1,2-diyl)dicarbamate (**21**). To a solution of compound **20** (4.4 mmol) in dry methanol (50 mL) was added Pd/C (10% w/w) and a 1 N aqueous HCl solution (2 equiv). The mixture was placed under H_2_ and stirred for 24 h at rt. After filtering the catalyst and evaporating methanol, the residue was dissolved in 1,4-dioxane (20 mL) before adding Boc_2_O (1.6 equiv) and triethylamine (6.9 equiv). The mixture was stirred for further 2 h at rt. The solvent was removed under reduced pressure and the resulting product was extracted with EtOAc (2 × 40 mL). The organic phase was washed with brine (3 × 40 mL), dried over Na_2_SO_4_ and concentrated in vacuo to give compound **21** as a colorless oil (1.3 g, 75%). *ν*_max_: 3343, 2926, 2855, 1679, 1522, 1366, 1248, 1163, 1056, 857, 607 cm^−1^. ^1^H NMR (400 MHz, chloroform-*d*): *δ* 4.92 (br s, 1H), 4.63 (br s, 1H), 3.61 (t, *^3^J* = 6.6 Hz, 2H), 3.58–3.50 (m, 1H), 3.16–3.12 (m, 2H), 1.79 (br s, 1H), 1.54 (quint, *^3^J* = 6.6 Hz, 2H), 1.41 (s, 18H), 1.31–1.25 (m, 14H). ^13^C NMR (101 MHz, chloroform-*d*): *δ* 156.8, 156.5, 79.5 (2C), 63.1, 51.5, 45.2, 33.3, 33.0 (2C), 29.6 (3C), 28.6 (6C), 25.9 (2C). HRMS (ESI): *m/z* calculated for C_21_H_42_N_2_O_5_Na, [M + Na]^+^ 425.2991, found 425.2982.

General procedure for the synthesis of mesylates 17 and 22: Compound **16** or **21** (1.4–3 mmol) was dissolved in CH_2_Cl_2_ (15–30 mL) and the solution was cooled at 0 °C. Triethylamine (1.1 equiv) and mesyl chloride (1.1 equiv) were then added. The mixture was stirred at 0 °C for 2 h. After evaporating CH_2_Cl_2_, the resulting product was extracted with EtOAc (2 × 30–60 mL). The organic phase was washed with a 0,5 N HCl solution (2 × 30–60 mL), a 5% NaHCO_3_ solution (2 × 30–60 mL) and brine (2 × 30–60 mL), dried over Na_2_SO_4_ and concentrated in vacuo to give compound **17** or **22**.

5,6-bis((tert-butoxycarbonyl)amino)hexyl methanesulfonate (**17**). Compound **17** was obtained from compound **16** (1.4 mmol) according to the general procedure as a white solid (455 mg, 80%) after purification by column chromatography on silica gel (CH_2_Cl_2_/MeOH 98:2). mp 70 °C. *ν*_max_: 3363, 2980, 2936, 1682, 1519, 1443, 1349, 1245, 1162, 976, 828, 626 cm^−1^. ^1^H NMR (400 MHz, chloroform-*d*): *δ* 4.89 (br s, 1H), 4.70 (br s, 1H), 4.19 (t, *^3^J* = 6.4 Hz, 2H), 3.62–3.58 (m, 1H), 3.16–3.12 (m, 2H), 2.98 (s, 3H), 1.78–1.69 (m, 2H), 1.49–1.44 (m, 4H), 1.41 (s, 18H). Q-DEPT NMR (101 MHz, chloroform-*d*): *δ* 156.8 (np), 156.4 (np), 79.7 (np, 2C), 69.9 (np), 51.4, 44.8 (np), 37.6, 32.5 (np), 29.1 (np), 28.6 (6C), 22.1 (np). HRMS (ESI): *m/z* calculated for C_17_H_34_N_2_O_7_SNa, [M + Na]^+^ 433.1984, found 433.2000.

10,11-bis((tert-butoxycarbonyl)amino)undecyl methanesulfonate (**22**). Compound **22** was obtained from compound **21** (3 mmol) according to the general procedure as a white solid (1.3 g, 91%). mp 62 °C. *ν*_max_: 3362, 2927, 2857, 1685, 1523, 1352, 1251, 1165, 941, 831, 627 cm^−1^. ^1^H NMR (400 MHz, chloroform-*d*): *δ* 4.87 (br s, 1H), 4.58 (br s, 1H), 4.20 (t, *^3^J* = 6.6 Hz, 2H), 3.60–3.56 (m, 1H), 3.26–3.09 (m, 2H), 2.98 (s, 3H), 1.72 (quint, *^3^J* = 6.6 Hz, 2H), 1.42 (s, 18H), 1.37–1.26 (m, 14H). ^13^C NMR (101 MHz, chloroform-*d*): *δ* 156.7, 156.5, 79.5 (2C), 70.4, 51.5, 45.2, 37.6, 33.2, 29.6 (2C), 29.5, 29.3, 29.2, 28.6 (6C), 26.0, 25.6. HRMS (ESI): *m/z* calculated for C_22_H_44_N_2_O_7_SNa, [M + Na]^+^ 503.2767, found 503.2767. 

##### Synthesis of Precursor **5**

di-tert-butyl (11-azidoundecane-1,2-diyl)dicarbamate (**23**). Compound **22** (1.2 mmol) was dissolved in DMF (8 mL). Sodium azide (2.5 equiv) was then added and the mixture was heated overnight at 60 °C under argon. The solvent was removed and the residue was taken up in EtOAc (3 × 20 mL). The organic phase was washed with brine (3 × 20 mL), dried over Na_2_SO_4_ and concentrated *in vacuo*. The crude material was purified by column chromatography on silica gel (EtOAc/Cyclohexane 2:98) to give compound **23** as a white solid (420 mg, 82%). mp 56 °C. *ν*_max_: 3341, 2975, 2925, 2855, 2091, 1675, 1526, 1457, 1366, 1247, 1163, 880, 755, 634 cm^−1^. ^1^H NMR (400 MHz, chloroform-*d*): *δ* 4.88 (br s, 1H), 4.59 (br s, 1H), 3.60–3.56 (m, 1H), 3.23 (t, *^3^J* = 7.0 Hz, 2H), 3.16–3.12 (m, 2H), 1.56 (quint, *^3^J* = 7.1 Hz, 2H), 1.41 (s, 18H), 1.30-1.23 (m, 14H). Q-DEPT NMR (101 MHz, chloroform-*d*): *δ* 156.8 (np), 156.5 (np), 79.5 (np, 2C), 51.7 (np), 51.5, 45.2 (np), 33.3 (np), 29.9 (np), 29.7 (np), 29.6 (np), 29.3 (np), 29.1 (np), 28.6 (6C), 26.9 (np), 26.1 (np). HRMS (ESI): *m/z* calculated for C_21_H_41_N_5_O_4_Na, [M + Na]^+^ 450.3056, found 450.3052.

di-tert-butyl (11-aminoundecane-1,2-diyl)dicarbamate (**5**). To a solution of compound **23** (0.4 mmol) in dry methanol (20 mL) was added Pd/C (10% w/w). The mixture was placed under H_2_ and stirred for 2 h at rt. After filtering the catalyst, the methanol was evaporated to give compound **5** as a yellow oil (155 mg, 88%). *ν*_max_: 3342, 2976, 2927, 2856, 2090, 1684, 1520, 1365, 1248, 1165, 1069, 732, 630cm^−1^. ^1^H NMR (400 MHz, chloroform-*d*): *δ* 4.95 (br s, 1H), 4.65 (br s, 1H), 3.58–3.54 (m, 1H), 3.15–3.11 (m, 2H), 2.64 (t, *^3^J* = 7.0 Hz, 1H), 2.57 (t, *^3^J* = 7.4 Hz, 1H), 1.87 (br s, 2H), 1.55–1.42 (m, 2H), 1.40 (s, 18H), 1.30–1.20 (m, 14H). Q-DEPT NMR (101 MHz, chloroform-*d*): *δ* 156.8 (np), 156.5 (np), 79.4 (np, 2C), 51.5, 45.1 (np), 42.4 (np), 33.9 (np), 33.2 (np), 29.7 (2C, np), 28.6 (6C), 27.6 (np), 27.1 (np), 26.1 (np), 25.6 (np). HRMS (ESI): *m/z* calculated for C_21_H_44_N_3_O_4_, [M + H]^+^ 402.3332, found 402.3332.

##### Synthesis of Precursors **7** and **8**

General procedure: Compound **17** or **22** (1–1.3 mmol) was dissolved in acetone (15–20 mL) and sodium iodide (5 equiv) was added. The mixture was stirred at rt for 10–16 h. The solvent was removed and the residue was taken up in EtOAc (3 × 30–40 mL). The organic phase was washed with brine (3 × 30–40 mL), dried over Na_2_SO_4_ and concentrated in vacuo to give compound **7**.

di-tert-butyl (6-iodohexane-1,2-diyl)dicarbamate (**7**). Compound **7** was obtained from compound **17** (1 mmol) according to the general procedure (16 h) as a white solid (230 mg, 53%). mp 68 °C. *ν*_max_: 3359, 2978, 2921, 1680, 1521, 1445, 1366, 1246, 1155, 1049, 880, 635 cm^−1^. ^1^H NMR (400 MHz, chloroform-*d*): *δ* 4.84 (br s, 1H), 4.64 (br s, 1H), 3.63–3.59 (m, 1H), 3.16 (t, *^3^J* = 6.9 Hz, 2H), 1.86–1.77 (m, 2H), 1.58–1.49 (m, 4H), 1.43 (s, 18H), 1.39–1.32 (m, 2H). ^13^C NMR (101 MHz, chloroform-*d*): *δ* 156.8, 156.4, 79.7 (2C), 51.5, 45.0, 33.4, 32.2, 30.0, 28.7 (6C), 27.1. HRMS (ESI): *m/z* calculated for C_16_H_31_N_2_O_4_INa, [M + Na]^+^ 465.1226, found 465.1228.

di-tert-butyl (11-iodoundecane-1,2-diyl)dicarbamate (**8**). Compound **8** was obtained from compound **22** (1.3 mmol) according to the general procedure (10 h) as a white solid (518 mg, 79%). mp 69 °C. *ν*_max_: 3361, 2977, 2923, 2851, 1681, 1522, 1462, 1363, 1245, 1166, 869, 622 cm^−1^. ^1^H NMR (400 MHz, chloroform-*d*): *δ* 4.93 (br s, 1H), 4.66 (br s, 1H), 3.59–3.55 (m, 1H), 3.13 (t, *^3^J* = 7.2 Hz, 2H), 1.80–1.73 (m, 4H), 1.38 (s, 18H), 1.22–1.15 (m, 14H). ^13^C NMR (101 MHz, chloroform-*d*): *δ* 156.7, 156.4, 79.4 (2C), 51.5, 45.1, 33.7, 33.2, 30.7, 29.6 (2C), 29.5, 28.7 (2C), 28.6 (6C), 26.0. HRMS (ESI): *m/z* calculated for C_21_H_41_N_2_O_4_INa, [M + Na]^+^ 535.2009, found 535.2004.

#### 3.2.3. Pseudopeptidic Coupling of Diamine Building Blocks **2–4** and **6** with HOPO Ligands **9a-b** and **10a-b** in the Series **Ia-b**

The synthetic routes to prepare compounds **3**, **4**, **6**, **9b**, **10b**, **27b**, **28b** and **Ib-3** have already been described by our research group [8]. 

For compound **9a** and **10a**, synthetic pathways and NMR spectra were in full accordance with those reported in the literature [10,11]. 

General procedure for the synthesis of compounds **24a-c** and **26a-b**: To a solution of compound **9a** or **9b** (0.7–1.4 mmol) in CH_2_Cl_2_, THF or THF/DMF 8:1 to 11:1 (20–45 mL) cooled at 0 °C were added 1-ethyl-3-(3-dimethylaminopropyl)-carbodiimide hydrochloride (EDC, 1.2–1.4 equiv) and 1-hydroxybenzotriazole monohydrate (HOBt, 1–1.2 equiv). After pre-stirring at 0 °C for 30 min, compound **2**, **3**, **4** or **6** (1 equiv) and triethylamine (0–1.2 equiv) were added in the mixture that was further stirred at rt for 6–24 h. The solvent was evaporated under reduced pressure and the residue was diluted in EtOAc (2 × 50–100 mL). The organic phase was washed with brine (3 × 50–100 mL) or a 1 N KHCO_3_ solution (2 × 50–100 mL), a 5% NaHCO_3_ solution (2 × 50–100 mL) and brine (2 × 50–100 mL), dried over Na_2_SO_4_ and concentrated in vacuo to give compound **24a-c** or **26a-b**.

di-tert-butyl5-(2-(3-(benzyloxy)-2-methyl-4-oxopyridin-1(4*H*)-yl)acetamido)pentane-1,2-diyl)dicarbamate (**24a**). Compound **24a** was obtained by coupling compounds **9a** (1.4 mmol) and **3** according to the general procedure (THF/DMF 8:1, 24 h) as an off-white solid (520 mg, 65%) after purification by column chromatography on silica gel (EtOAc/MeOH 80:20). mp 101 °C. ^1^H NMR (300 MHz, methanol-*d_4_*): *δ* 7.61 (d, *^3^J* = 7.5 Hz, 1H), 7.41–7.31 (m, 5H), 6.46 (d, *^3^J* = 7.5 Hz, 1H), 5.05 (s, 2H), 4.68 (s, 2H), 3.58–3.54 (m, 1H), 3.23–3.18 (m, 2H), 3.12–2.97 (m, 2H), 2.07 (s, 3H), 1.58–1.30 (m, 22H). Q-DEPT NMR (75 MHz, methanol-*d_4_*): *δ* 175.6 (np), 168.5 (np), 163.7 (np), 158.7 (np), 147.3 (np), 145.9 (np), 143.0, 138.8 (np), 130.4 (2C), 129.8 (2C), 129.6, 117.4, 80.4 (np), 80.3 (np), 75.0 (np), 57.2 (np), 52.2, 45.8 (np), 40.9 (np), 31.2 (np), 29.1 (6C), 27.2 (np), 13.3. HRMS (ESI): *m/z* calculated for C_30_H_44_N_4_O_7_Na, [M + Na]^+^ 595.3108, found 595.3121.

di-tert-butyl(6-(2-(3-(benzyloxy)-2-methyl-4-oxopyridin-1(4*H*)-yl)acetamido)hexane-1,2-diyl)dicarbamate (**24b**). Compound **24b** was obtained by coupling compounds **9a** (1 mmol) and **4** according to the general procedure (THF/DMF 8:1, 24 h) as an off-white solid (364 mg, 62%) after purification by column chromatography on silica gel (EtOAc/MeOH 80:20). mp 79 °C. *ν*_max_: 1680, 1519, 1364, 1249, 1162, 999, 746 cm^−1^. ^1^H NMR (300 MHz, methanol-*d_4_*): *δ* 7.62 (d, *^3^J* = 7.2 Hz, 1H), 7.42–7.32 (m, 5H), 6.47 (d, *^3^J* = 7.2 Hz, 1H), 5.06 (s, 2H), 4.68 (s, 2H), 3.56–3.54 (m, 1H), 3.23–3.18 (m, 2H), 3.14–2.98 (m, 2H), 2.08 (s, 3H), 1.49–1.35 (m, 24H). Q-DEPT NMR (75 MHz, methanol-*d_4_*): *δ* 175.3 (np), 168.2 (np), 161.1 (np), 158.7 (np), 147.0 (np), 145.6 (np), 142.7, 138.5 (np), 130.2 (2C), 129.5 (2C), 129.3, 117.1, 80.1 (np), 80.0 (np), 74.7 (np), 56.9 (np), 52.1, 45.5 (np), 40.6 (np), 33.1 (np), 30.1 (np), 28.9 (6C), 24.4 (np), 13.0. HRMS (ESI): *m/z* calculated for C_31_H_46_N_4_O_7_Na, [M + Na]^+^ 609.3264, found 609.3271.

di-tert-butyl(5-(4-(2-(3-(benzyloxy)-2-methyl-4-oxopyridin-1(4*H*)-yl)acetyl)piperazin-1-yl)-5-oxopentane -1,2-diyl)dicarbamate (**24c**). Compound **24c** was obtained by coupling compounds **9a** (1 mmol) and **6** according to the general procedure (CH_2_Cl_2_, 18 h) as a white solid (348 mg, 51%) after purification by column chromatography on silica gel (CH_2_Cl_2_/MeOH 95:5). mp 105 °C. *ν*_max_: 3333, 2976, 1698, 1629, 1511, 1434, 1245, 1163, 1018, 742 cm^−1^. ^1^H NMR (400 MHz, chloroform-*d*): *δ* 7.35–7.28 (m, 6H), 6.42 (d, *^3^J* = 7.2 Hz, 1H), 5.17 (s, 2H), 5.03 (s, 2H), 4.83 (br s, 2H), 3.60–3.42 (m, 9H), 3.21–3.17 (m, 2H), 2.43–2.37 (m, 2H), 2.04 (s, 3H), 1.88–1.67 (m, 2H), 1.43 (s, 18H). Q-DEPT NMR (101 MHz, chloroform-*d*): *δ* 173.1 (np), 171.4 (np), 164.5 (np), 157.0 (np), 156.3 (np), 145.6 (np), 144.1 (np), 140.8, 137.6 (np), 129.0 (2C), 128.7 (2C), 128.4, 116.3, 79.6 (np, 2C), 73.8 (np), 55.3 (np), 51.6, 45.1 (np), 44.7 (np), 42.5 (np), 41.7 (np), 41.4 (np), 29.7 (np), 28.6 (6C), 28.0 (np), 13.0. HRMS (ESI): *m/z* calculated for C_34_H_49_N_5_O_8_Na, [M + Na]^+^ 678.3479, found 678.3464.

benzyl(1-azido-4-(2-(3-(benzyloxy)-2-methyl-4-oxopyridin-1(4*H*)-yl)acetamido)butan-2-yl)carbamate (**26a**). Compound **26a** was obtained by coupling compounds **9a** (0.7 mmol) and **2** according to the general procedure (THF/DMF 11:1, 6 h) as a yellow pasty solid (312 mg, 86%) after purification by column chromatography on silica gel (EtOAc/MeOH 50:50). *ν*_max_: 3263, 3034, 2098, 1678, 1626, 1549, 1254, 1064, 738, 698 cm^−1^. ^1^H NMR (400 MHz, methanol-*d_4_*): *δ* 7.57 (d, *^3^J* = 7.4 Hz, 1H), 7.36 (d, *^3^J* = 7.7 Hz, 2H), 7.31–7.25 (m, 8H), 6.42 (d, *^3^J* = 7.4 Hz, 1H), 5.03 (s, 2H), 5.00 (s, 2H), 4.63 (s, 2H), 3.75–3.72 (m, 1H), 3.31–3.21 (m, 4H), 2.05 (s, 3H), 1.72–1.56 (m, 2H). Q-DEPT NMR (101 MHz, methanol-*d_4_*): *δ* 173.9 (np), 166.9 (np), 157.2 (np), 145.6 (np), 144.2 (np), 141.5, 137.1 (np), 137.0 (np), 128.7 (3C), 128.1 (2C), 127.9 (2C), 127.6, 127.3 (2C), 115.7, 73.3 (np), 66.2 (np), 55.4 (np), 54.4 (np), 48.8, 35.9 (np), 31.1 (np), 11.9. HRMS (ESI): *m/z* calculated for C_27_H_30_N_6_O_5_Na, [M + Na]^+^ 541.2175, found 541.2186.

benzyl(1-azido-4-(3-(3-(benzyloxy)-2-methyl-4-oxopyridin-1(4*H*)-yl)propanamido)butan-2-yl)carbamate (**26b**). Compound **26b** was obtained by coupling compounds **9b** (1.1 mmol) and **2** according to the general procedure (THF, 6 h) as a yellow pasty solid (410 mg, 70%) after purification by column chromatography on silica gel (EtOAc/MeOH 50:50). *ν*_max_: 3262, 3063, 2942, 2097, 1623, 1548, 1246, 1062, 735, 698 cm^−1^. ^1^H NMR (400 MHz, chloroform-*d*): *δ* 7.89 (br s, 1H), 7.30–7.24 (m, 10H), 7.17 (d, *^3^J* = 7.5 Hz, 1H), 6.21 (d, *^3^J* = 7.4 Hz, 1H), 5.92 (br s, 1H), 5.04 (s, 2H), 5.01 (s, 2H), 4.00–3.96 (m, 2H), 3.71–3.67 (m, 1H), 3.37–3.32 (m, 2H), 3.30–3.05 (m, 2H), 2.37–2.34 (m, 2H), 2.06 (s, 3H), 1.66–1.63 (m, 2H). Q-DEPT NMR (101 MHz, chloroform-*d*): *δ* 173.2 (np), 168.9 (np), 156.4 (np), 145.8 (np), 141.9 (np), 139.1, 137.2 (np), 137.1 (np), 128.9 (2C), 128.5 (3C), 128.4 (2C), 128.2 (2C), 128.1, 116.9, 73.4 (np), 68.8 (np), 54.8 (np), 50.1 (np), 49.1, 36.8 (np), 35.8 (np), 31.7 (np), 12.3. HRMS (ESI): *m/z* calculated for C_28_H_32_N_6_O_5_Na, [M + Na]^+^ 555.2332, found 555.2332.

di-tert-butyl(5-(4-(2-(3-(benzyloxy)-2-oxopyridin-1(2*H*)-yl)acetyl)piperazin-1-yl)-5-oxopentane-1,2-diyl) dicarbamate (**27a**). To a solution of compound **10a** (1.9 mmol) in 1,4-dioxane (25 mL) were added NHS (1.5 equiv) and DCC (1.2 equiv). The mixture was stirred overnight at rt. After filtering off DCC urea and evaporating 1,4-dioxane, the activated intermediate (1.2 mmol) was added with a pre-stirred solution of compound 6 (1.2 equiv) and triethylamine (3 equiv) in CH_2_Cl_2_ (25 mL). The mixture was stirred at rt for 48 h and diluted with additional CH_2_Cl_2_ (2 × 25 mL). The organic phase was washed with 1 N HCl (2 × 25 mL), saturated NaHCO_3_ solution (2 × 25 mL) and brine (3 × 25 mL), dried over Na_2_SO_4_ and concentrated *in vacuo* to give compound **27a** as a white solid (448 mg, 56%) yield after purification by column chromatography on silica gel (CH_2_Cl_2_/MeOH 95:5). mp 97 °C. *ν*_max_: 3324, 2978, 2936, 2851, 1697, 1651, 1601, 1436, 1247, 1163, 1046, 741 cm^−1^. ^1^H NMR (400 MHz, chloroform-*d*): *δ* 7.38 (d, *^3^J* = 7.1 Hz, 2H), 7.32 (t, *^3^J* = 7.5 Hz, 2H), 7.26 (t, *^3^J* = 7.8 Hz, 1H), 7.27 (m, 1H), 6.65 (d, *^3^J* = 7.5 Hz, 1H), 6.05 (t, *^3^J* = 7.0 Hz, 1H), 5.06 (s, 2H), 5.03 (br s, 2H), 4.76–4.69 (m, 2H), 3.75–3.41 (m, 9H), 3.17–3.14 (m, 2H), 2.40–2.34 (m, 2H), 1.85–1.63 (m, 2H), 1.39 (s, 18H). Q-DEPT NMR (101 MHz, chloroform-*d*): *δ* 171.3 (np), 165.6 (np, 2C), 158.0 (np), 156.1 (np), 148.3 (np), 135.9 (np), 129.6, 128.5 (2C), 127.9, 127.2 (2C), 115.9, 104.8, 79.3 (np, 2C), 70.7 (np), 51.4, 49.2 (np), 45.2 (np), 44.8 (np), 44.4 (np), 42.0 (np), 41.4 (np), 29.7 (np), 28.3 (6C), 27.8 (np). HRMS (ESI): *m/z* calculated for C_33_H_47_N_5_O_8_Na, [M + Na]^+^ 664.3322, found 664.3324.

#### 3.2.4. Condensation of Maltol Benzyl Ether with Amino Starting Blocks **4** and **5** in the Series **IIa**

For maltol benzyl ether, synthetic procedure and NMR spectra were in full accordance with those reported in the literature [10,12]. 

General procedure: To a solution of NaOH (0.5–2.6 equiv) in EtOH/H_2_O 1:1 to 1:2 (5–120 mL) were added compound **4** or **5** (0.3–3.4 mmol) and maltol benzyl ether (1.1–1.5 equiv). The mixture was heated under reflux for 18–24 h. After evaporating ethanol under reduced pressure, the aqueous phase was neutralized with a 6 N HCl solution and the resulting product was extracted with EtOAc (3 × 20–250 mL). The organic phase was washed with brine (3 × 20–250 mL), dried over Na_2_SO_4_ and concentrated in vacuo to give compound **24d** or **24e**.

di-tert-butyl (6-(3-(benzyloxy)-2-methyl-4-oxopyridin-1(4*H*)-yl)hexane-1,2-diyl)dicarbamate (**24d**). Compound **24d** was obtained by coupling compound **4** (3.4 mmol) and maltol benzyl ether (1.5 equiv) according to the general procedure (NaOH (0.5 equiv), EtOH/H_2_O 1:1, 24 h) as a yellow pasty solid (1.1 g, 61%) after purification by column chromatography on silica gel (EtOAc/MeOH 90:10). ^1^H NMR (400 MHz, chloroform-*d*): *δ* 7.38 (d, *^3^J* = 6.7 Hz, 2H), 7.32–7.26 (m, 3H), 7.16 (d, *^3^J* = 7.5 Hz, 1H), 6.39 (d, *^3^J* = 7.5 Hz, 1H), 5.03 (s, 2H), 4.94 (br s, 2H), 3.71–3.76 (m, 2H), 3.61–3.57 (m, 1H), 3.163.12 (m, 2H), 2.06 (s, 3H), 1.66–1.55 (m, 2H), 1.40–1.35 (m, 22H). Q-DEPT NMR (101 MHz, chloroform-*d*): *δ* 173.5 (np), 156.6 (np, 2C), 146.4 (np), 141.0 (np), 138.3, 137.9 (np), 129.3 (2C), 128.4 (2C), 128.1, 117.5, 79.7 (np, 2C), 73.1 (np), 53.9 (np), 51.2, 44.7 (np), 32.6 (np), 30.9 (np), 28.5 (6C), 23.0 (np), 12.5. HRMS (ESI): *m/z* calculated for C_29_H_44_N_3_O_6_, [M + H]^+^ 530.3230, found 530.3245.

di-tert-butyl(11-(3-(benzyloxy)-2-methyl-4-oxopyridin-1(4*H*)-yl)undecane-1,2-diyl)dicarbamate (**24e**). Compound **24e** was obtained by coupling compound **5** (0.3 mmol) and maltol benzyl ether (1.1 equiv) according to the general procedure (NaOH (2.6 equiv), EtOH/H_2_O 1:2, 18 h) as an orange oil (113 mg, 63%) after purification by column chromatography on silica gel (EtOAc/MeOH 90:10). *ν*_max_: 2975, 2928, 2856, 1695, 1625, 1514, 1364, 1245, 1164, 1061, 730, 625 cm^−1^. ^1^H NMR (400 MHz, chloroform-*d*): *δ* 7.38 (dd, *^3^J* = 7.6 Hz, *^4^J* = 1.8 Hz, 2H), 7.29–7.26 (m, 3H), 7.16 (d, *^3^J* = 7.5 Hz, 1H), 6.40 (d, *^3^J* = 7.5 Hz, 1H), 5.20 (s, 2H), 4.96 (br s, 1H), 4.70 (br s, 1H), 3.69 (t, *^3^J* = 6.1 Hz, 2H), 3.60–3.56 (m, 1H), 3.17–3.13 (m, 2H), 2.05 (s, 3H), 1.62-1.57 (m, 2H), 1.41 (s, 18H), 1.34–1.23 (m, 14H). ^13^C NMR (101 MHz, chloroform-*d*): *δ* 173.5, 156.8, 156.5, 146.3, 140.8, 138.3, 137.9, 129.4 (2C), 128.4 (2C), 128.2, 117.5, 79.5 (2C), 73.2, 54.1, 51.5, 45.1, 33.2, 31.0, 29.6, 29.5, 29.4, 29.3, 28.6 (6C), 26.5, 26.0, 12.6. HRMS (ESI): *m/z* calculated for C_34_H_54_N_3_O_6_, [M + H]^+^ 600.4013, found 600.4024.

#### 3.2.5. Nucleophilic Substitution of Iodo Starting Blocks **7** and **8** by 3,2-HOPO Benzyl Ether in the Series **IIa**

For 3,2-HOPO benzyl ether, synthetic procedure and NMR spectra were in full accordance with those reported in the literature [13]. 

General procedure: To a solution of 3,2-HOPO benzyl ether (0.3–0.6 mmol) in DMF (2–3 mL) cooled at 0 °C under argon was added a NaH 60% dispersion in mineral oil (1 equiv). After stirring at rt for 20 min, the mixture was cooled back to 0 °C and compound **7** or **8** (1 equiv) was introduced. The mixture was further stirred at rt for 2 h. The reaction was quenched with a saturated aqueous NH_4_Cl solution (5–10 mL). After evaporating DMF, the resulting product was extracted with EtOAc (3 × 15 mL). The organic phase was washed with brine (3 × 15 mL), dried over Na_2_SO_4_ and concentrated *in vacuo* to give compound **27c** or **27d**.

di-tert-butyl (6-(3-(benzyloxy)-2-oxopyridin-1(2*H*)-yl)hexane-1,2-diyl)dicarbamate (**27c**). Compound **27c** was obtained by coupling 3,2-HOPO benzyl ether (0.3 mmol) and compound **7** according to the general procedure as a yellow oil (93 mg, 72%) after purification by column chromatography on silica gel (CH_2_Cl_2_/MeOH 95:5). *ν*_max_: 3316, 2974, 2930, 1697, 1651, 1599, 1514, 1365, 1248, 1169, 1057, 911, 734, 634 cm^−1^. ^1^H NMR (400 MHz, chloroform-*d*): *δ* 7.34 (d, *^3^J* = 7.3 Hz, 2H), 7.26 (t, *^3^J* = 6.1 Hz, 2H), 7.20 (t, *^3^J* = 7.2 Hz, 1H), 6.80 (dd, *^3^J* = 6.9 Hz, *^4^J* = 1.6 Hz, 1H), 6.55 (dd, *^3^J* = 7.4 Hz, *^4^J* = 1.6 Hz, 1H), 5.92 (t, *J* = 7.1 Hz, 1H), 5.01 (s, 2H), 4.99 (br s, 1H), 4.82 (br s, 1H), 3.86 (t, *J* = 7.1 Hz, 2H), 3.53–3.49 (m, 1H), 3.09–3.05 (m, 2H), 1.69–1.66 (m, 2H), 1.52–1.43 (m, 2H), 1.33 (s, 18H), 1.32–1.25 (m, 2H). ^13^C NMR (101 MHz, chloroform-*d*): *δ* 158.2, 156.8, 156.4, 149.0, 136.5, 129.1, 128.6 (2C), 128.1, 127.4 (2C), 115.7, 104.8, 79.4 (2C), 70.8, 51.4, 49.6, 44.7, 32.5, 29.1, 28.5 (6C), 23.0. HRMS (ESI): *m/z* calculated for C_28_H_41_N_3_O_6_Na, [M + Na]^+^ 538.2893, found 538.2889.

di-tert-butyl (11-(3-(benzyloxy)-2-oxopyridin-1(2*H*)-yl)undecane-1,2-diyl)dicarbamate (**27d**). Compound **27d** was obtained by coupling 3,2-HOPO benzyl ether (0.6 mmol) and compound **8** according to the general procedure as an off-white solid (232 mg, 62%) after purification by column chromatography on silica gel (EtOAc/Cyclohexane 80:20). mp 72 °C. *ν*_max_: 3360, 2980, 2922, 2853, 1682, 1649, 1595, 1525, 1456, 1367, 1239, 1163, 1051, 751, 647 cm^−1^. ^1^H NMR (400 MHz, chloroform-*d*): *δ* 7.41 (d, *^3^J* = 7.3 Hz, 2H), 7.32 (t, *^3^J* = 7.2 Hz, 2H), 7.26 (t, *^3^J* = 7.2 Hz, 1H), 6.86 (dd, *^3^J* = 6.9 Hz, *^4^J* = 1.7 Hz, 1H), 6.61 (dd, *^3^J* = 7.3 Hz, *^4^J* = 1.1 Hz, 1H), 5.98 (t, *^3^J* = 6.7 Hz, 1H), 5.09 (s, 2H), 4.92 (br s, 1H), 4.62 (br s, 1H), 3.92 (t, *^3^J* = 2.8 Hz, 2H), 3.59–3.55 (m, 1H), 3.16–3.12 (m, 2H), 1.74–1.70 (m, 2H), 1.41 (s, 20H), 1.28–1.23 (m, 12H). ^13^C NMR (101 MHz, chloroform-*d*): *δ* 158.3, 156.7, 156.4, 149.1, 136.7, 129.3, 128.7 (2C), 128.1, 127.5 (2C), 115.7, 104.7, 79.4 (2C), 70.9, 50.1, 45.1, 33.2, 29.6, 29.5 (2C), 29.4, 29.3, 28.8, 28.6 (6C), 26.8, 26.0. HRMS (ESI): *m/z* calculated for C_33_H_51_N_3_O_6_Na, [M + Na]^+^ 608.3676, found 608.3682. 

#### 3.2.6. Synthesis of Piperazine-1,4-Dialkyl Intermediate **27e** from Piperazine-1,4-Diamide Analog **27b** in the Series **IIb**

di-tert-butyl(5-(4-(3-(3-(benzyloxy)-2-oxopyridin-1(2*H*)-yl)propyl)piperazin-1-yl)pentane-1,2-diyl) dicarbamate (**27e**). To a solution of compound **27b** (1 mmol) in THF (100 mL) was added dropwise a 2 M BH_3_.Me_2_S solution (5 equiv) in THF. The mixture was heated under reflux for 2 h. After cooling to rt, the reaction was quenched with methanol (50 mL) and the mixture was further heated under reflux for 18 h. The solvents were evaporated and the residue was taken up in EtOH/1 N NaOH 5:1 (30 mL). After heating under reflux for 2 h, the ethanol was removed under reduced pressure and the aqueous phase was extracted with EtOAc (3 × 50 mL). The organic phase was washed with brine (3 × 50 mL), dried over Na_2_SO_4_ and concentrated in vacuo to give compound **27e** as a colorless oil (276 mg, 44%) after purification by column chromatography on silica gel (EtOAc/MeOH 50:50). *ν*_max_: 3330, 2933, 1691, 1650, 1515, 1364, 1246, 1161, 739 cm^−1^. ^1^H NMR (400 MHz, chloroform-*d*): *δ* 7.40 (d, *^3^J* = 7.3 Hz, 2H), 7.32 (t, *^3^J* = 7.2 Hz, 2H), 7.26 (t, *^3^J* = 7.8 Hz, 1H), 6.93 (d, *^3^J* = 6.8 Hz, 1H), 6.61 (d, *^3^J* = 7.4 Hz, 1H), 5.96 (t, *^3^J* = 7.1 Hz, 1H), 5.27 (s, 2H), 5.25 (br s, 1H), 5.10–5.06 (m, 2H), 4.98 (br s, 1H), 3.99 (t, *^3^J* = 6.8 Hz, 2H), 3.59–3.55 (m, 1H), 3.16–3.12 (m, 2H), 2.43–2.31 (m, 10H), 1.92 (t, *^3^J* = 6.8 Hz, 2H), 1.54–1.52 (m, 2H), 1.40 (m, 20H). Q-DEPT NMR (101 MHz, chloroform-*d*): *δ* 158.5 (np, 2C), 149.0 (np), 136.8 (np), 129.8, 128.7 (2C), 128.1, 127.5 (2C), 115.7, 104.5, 79.4 (2C), 70.9 (np), 58.2 (np), 54.8 (np, 2C), 53.4 (np), 53.0 (np), 51.2, 48.2 (np), 45.1 (np), 30.7 (np), 29.9 (np), 28.6 (6C), 26.0 (np), 23.2 (np). HRMS (ESI): *m/z* calculated for C_34_H_54_N_5_O_6_, [M + H]^+^ 628.4074, found 628.4080. 

#### 3.2.7. Debenzylation of Hydroxy Group of Derivatives **24a-e** and **27a-e** and Synthesis of Final Products **Ia-3** and **Ia-4** by Catalytic Hydrogenation

General procedure: To a solution of compound **24a-e**, **27a-e** or **26a-b** (0.2–1.4 mmol) in dry methanol (5–20 mL) was added Pd/C or Pd(OH)_2_/C (10% w/w). The mixture was placed under H_2_ and stirred for 3–48 h at rt. After filtering the catalyst, the methanol was evaporated to give compound **25a-e**, **28a-e**, **Ia-3** or **Ia-4**.

di-tert-butyl(5-(2-(3-hydroxy-2-methyl-4-oxopyridin-1(4*H*)-yl)acetamido)pentane-1,2-diyl)dicarbamate (**25a**). Compound **25a** was obtained from compound **24a** (0.7 mmol) according to the general procedure (Pd/C, 6 h) as a white solid (263 mg, 78%). mp 138 °C. *ν*_max_: 3350, 2976, 1682, 1508, 1246, 1162, 666 cm^−1^. ^1^H NMR (400 MHz, chloroform-*d*): *δ* 7.49 (d, *^3^J* = 4.8 Hz, 1H), 6.33 (d, *^3^J* = 4.8 Hz, 1H), 4.70 (s, 2H), 3.53–3.51 (m, 1H), 3.19–3.17 (m, 2H), 3.07–2.91 (m, 2H), 2.26 (s, 3H), 1.561.44 (m, 4H), 1.42 (s, 18H). Q-DEPT NMR (400 MHz, chloroform-*d*): *δ* 169.9 (np), 167.1 (np), 157.3 (np), 157.0 (np), 145.5 (np), 139.0, 131.9 (np), 111.1, 78.6 (np, 2C), 55.5 (np), 50.5, 44.1 (np), 39.2 (np), 29.6 (np), 27.4 (6C), 25.4 (np), 10.7. HRMS (ESI): *m/z* calculated for C_23_H_38_N_4_O_7_Na, [M + Na]^+^ 505.2638, found 505.2636.

di-tert-butyl(6-(2-(3-hydroxy-2-methyl-4-oxopyridin-1(4*H*)-yl)acetamido)hexane-1,2-diyl)dicarbamate (**25b**). Compound **25b** was obtained from compound **24b** (0.4 mmol) according to the general procedure (Pd/C, 6 h) as a white solid (141 mg, 71%). mp 115 °C. *ν*_max_: 3346, 2979, 2934, 1676, 1510, 1245, 1161, 653 cm^−1^. ^1^H NMR (400 MHz, chloroform-*d*): *δ* 7.57 (d, *^3^J* = 4.8 Hz, 1H), 6.41 (d, *^3^J* = 4.8 Hz, 1H), 4.77 (s, 2H), 3.59–3.55 (m, 1H), 3.14–3.11 (m, 2H), 3.02–2.98 (m, 2H), 2.34 (s, 3H), 1.58–1.49 (m, 2H), 1.42–1.38 (m, 20H), 1.39–1.35 (m, 2H). Q-DEPT NMR (400 MHz, chloroform-*d*): *δ* 169.9 (np), 167.1 (np), 157.3 (np), 157.0 (np), 145.5 (np), 139.0, 131.9 (np), 111.1, 78.6 (np, 2C), 55.5 (np), 50.7, 44.0 (np), 39.1 (np), 31.6 (np), 28.7 (np), 27.4 (6C), 22.9 (np), 10.7. HRMS (ESI): *m/z* calculated for C_24_H_40_N_4_O_7_Na, [M + Na]^+^ 519.2795, found 519.2789.

di-tert-butyl(5-(4-(2-(3-hydroxy-2-methyl-4-oxopyridin-1(4*H*)-yl)acetyl)piperazin-1-yl)-5-oxopentane -1,2-diyl)dicarbamate (**25c**) Compound **25c** was obtained from compound **24c** (0.4 mmol) according to the general procedure (Pd/C, 48 h) as a white solid (218 mg, 100%). mp 96 °C. *ν*_max_: 3295, 1631, 1511, 1435, 1363, 1243, 1163, 1017, 753 cm^−1^. ^1^H NMR (400 MHz, chloroform-*d*): *δ* 7.31–7.27 (m, 1H), 6.37–6.33 (m, 1H), 5.98 (br s, 2H), 5.13 (br s, 1H), 4.88 (s, 2H), 3.67–3.43 (m, 9H), 3.16–3.12 (m, 2H), 2.37–2.33 (m, 2H), 2.17 (s, 3H), 1.80–1.63 (m, 2H), 1.40 (s, 18H). Q-DEPT NMR (101 MHz, chloroform-*d*): *δ* 171.6 (np), 168.9 (np), 168.6 (np), 164.5 (np), 156.9 (np), 142.8 (np), 138.6, 128.5 (np), 111.5, 79.6 (np, 2C), 55.0 (np), 50.7, 45.2 (np), 44.9 (np), 44.6 (np), 42.1 (np), 41.5 (np), 29.9 (np), 28.4 (6C), 28.1 (np), 12.2. HRMS (ESI): *m/z* calculated for C_27_H_43_N_5_O_8_Na, [M + Na]^+^ 588.3009, found 588.3002.

di-tert-butyl (6-(3-hydroxy-2-methyl-4-oxopyridin-1(4*H*)-yl)hexane-1,2-diyl)dicarbamate (**25d**). Compound **25d** was obtained from compound **24d** (1.4 mmol) according to the general procedure (Pd/C, 8 h) as an orange solid (560 mg, 91%). mp 60 °C. *ν*_max_: 3318, 2975, 1687, 1506, 1363, 1242, 1161, 1038, 826 cm^−1^. ^1^H NMR (400 MHz, chloroform-*d*): *δ* 7.88–7.84 (m, 1H), 7.03–7.00 (m, 2H), 5.19 (br s, 1H), 5.11 (br s, 1H), 4.21–4.17 (m, 2H), 3.61–3.57 (m, 1H), 3.15–3.11 (m, 2H), 2.51 (s, 3H), 1.84–1.78 (m, 2H), 1.40–1.37 (m, 22H). ^13^C NMR (101 MHz, chloroform-*d*): *δ* 161.5, 157.1, 156.6, 144.5, 138.4, 137.5, 112.3, 79.6 (2C), 56.3, 51.2, 44.6, 32.3, 30.3, 28.6 (6C), 22.8, 12.8. HRMS (ESI): *m/z* calculated for C_22_H_38_N_3_O_6_, [M + H]^+^ 440.2761, found 440.2749.

di-tert-butyl (11-(3-hydroxy-2-methyl-4-oxopyridin-1(4*H*)-yl)undecane-1,2-diyl)dicarbamate (**25e**). Compound **25e** was obtained from compound **24e** (0.2 mmol) according to the general procedure (Pd/C, 4 h) as a yellow oil (64 mg, 84%). *ν*_max_: 2928, 2855, 1694, 1575, 1507, 1454, 1363, 1242, 1162, 1036, 830, 776, 670, 629 cm^−1^. ^1^H NMR (400 MHz, chloroform-*d*): *δ* 7.29 (d, *^3^J* = 5.6 Hz, 1H), 6.45 (d, *^3^J* = 7.6 Hz, 1H), 5.49 (br s, 1H), 5.08 (br s, 1H), 4.80 (br s, 1H), 3.88 (t, *^3^J* = 7.9 Hz, 2H), 3.64–3.60 (m, 1H), 3.19–3.15 (m, 2H), 2.40 (s, 3H), 1.74–1.59 (m, 2H), 1.43 (s, 18H), 1.31–1.25 (m, 14H). ^13^C NMR (101 MHz, chloroform-*d*): *δ* 169.0, 156.5, 156.3, 146.4, 136.9, 129.1, 111.5, 79.5 (2C), 54.4, 51.4, 45.0, 33.2, 31.1, 29.5, 29.4 (2C), 29.2, 28.6 (6C), 26.5, 26.0, 12.2. HRMS (ESI): *m/z* calculated for C_27_H_48_N_3_O_6_, [M + H]^+^ 510.3543, found 510.3540.

di-tert-butyl(5-(4-(2-(3-hydroxy-2-oxopyridin-1(2*H*)-yl)acetyl)piperazin-1-yl)-5-oxopentane-1,2-diyl)dicarbamate (**28a**). Compound **28a** was obtained from compound **27a** (0.5 mmol) according to the general procedure (Pd/C, 48 h) as an off-white solid (246 mg, 95%). mp 104 °C. *ν*_max_: 3333, 2933, 1648, 1598, 1601, 1439, 1246, 1161, 1027, 871, 740, 607 cm^−1^. ^1^H NMR (400 MHz, chloroform-*d*): *δ* 6.82–6.78 (m, 2H), 6.18 (t, *^3^J* = 7.1 Hz, 1H), 5.03 (br s, 2H), 4.854.71 (m, 2H), 3.86–3.38 (m, 10H), 3.21–3.15 (m, 2H), 2.46–2.32 (m, 2H), 1.92–1.64 (m, 2H), 1.40 (s, 18H). Q-DEPT NMR (101 MHz, chloroform-*d*): *δ* 171.6 (np), 165.4 (np), 158.9 (np), 157.2 (np), 156.6 (np), 146.7 (np), 128.1, 114.9, 107.2, 79.8 (np, 2C), 51.6, 49.9 (np), 45.2 (np), 44.8 (np), 42.5 (np), 41.5 (np), 34.1 (np), 30.0 (np), 28.6 (6C), 28.2 (np). HRMS (ESI): *m/z* calculated for C_26_H_41_N_5_O_8_Na, [M + Na]^+^ 574.2853, found 574.2832.

di-tert-butyl (6-(3-hydroxy-2-oxopyridin-1(2*H*)-yl)hexane-1,2-diyl)dicarbamate (**28c**). Compound **28c** was obtained from compound **27c** (0.2 mmol) according to the general procedure (Pd/C, 3 h) as a yellow pasty solid (56 mg, 66%). *ν*_max_: 3348, 2978, 2931, 1680, 1591, 1525, 1243, 1161, 1061, 738, 643 cm^−1^. ^1^H NMR (400 MHz, chloroform-*d*): *δ* 6.79 (d, *^3^J* = 7.1 Hz, 2H), 6.14 (t, *^3^J* = 7.1 Hz, 1H), 5.02 (br s, 1H), 4.91 (br s, 1H), 4.05–3.92 (m, 2H), 3.60–3.56 (m, 1H), 3.18–3.14 (m, 2H), 1.81–1.74 (m, 2H), 1.73–1.50 (m, 2H), 1.44–1.40 (m, 20H). ^13^C NMR (101 MHz, chloroform-*d*): *δ* 158.9 (2C), 156.5, 146.8, 127.1, 113.8, 107.2, 79.6 (2C), 51.4, 49.4, 45.0, 30.0, 29.4, 28.7 (6C), 22.8. HRMS (ESI): *m/z* calculated for C_21_H_35_N_3_O_6_Na, [M + Na]^+^ 448.2424, found 448.2426.

di-tert-butyl (11-(3-hydroxy-2-oxopyridin-1(2*H*)-yl)undecane-1,2-diyl)dicarbamate (**28d**). Compound **28d** was obtained from compound **27d** (0.3 mmol) according to the general procedure (Pd/C, 3 h) as a brown oil (103 mg, 78%). *ν*_max_: 3344, 2926, 2856, 1690, 1596, 1516, 1365, 1245, 1162, 1052, 755 cm^−1^. ^1^H NMR (400 MHz, chloroform-*d*): *δ* 6.80–6.76 (m, 2H), 6.13 (t, *^3^J* = 7.2 Hz, 1H), 4.95 (br s, 1H), 4.69 (br s, 1H), 3.97–3.93 (m, 2H), 3.60–3.56 (m, 1H), 3.16–3.12 (m, 2H), 1.75–1.71 (m, 2H), 1.43–1.39 (m, 26H), 1.31–1.27 (m, 6H). ^13^C NMR (101 MHz, chloroform-*d*): *δ* 158.7, 156.8, 156.5, 146.8, 127.2, 113.8, 106.9, 79.5 (2C), 51.5, 50.3, 45.2, 33.2, 29.7, 29.6 (2C), 29.5, 29.4, 29.3, 28.6 (6C), 26.7, 26.0. HRMS (ESI): *m/z* calculated for C_26_H_45_N_3_O_6_Na, [M + Na]^+^ 518.3206, found 518.3207.

di-tert-butyl(5-(4-(3-(3-hydroxy-2-oxopyridin-1(2*H*)-yl)propyl)piperazin-1-yl)pentane-1,2-diyl)dicarbamate (**28e**). Compound **28e** was obtained from compound **27e** (0.4 mmol) according to the general procedure (Pd/C, 6 h) as a brown pasty solid (202 mg, 94%). *ν*_max_: 3330, 2975, 1686, 1517, 1364, 1246, 1161, 752 cm^−1^. ^1^H NMR (400 MHz, chloroform-*d*): *δ* 6.83 (d, *^3^J* = 6.8 Hz, 1H), 6.76 (d, *^3^J* = 7.3 Hz, 1H), 6.11 (t, *^3^J* = 7.1 Hz, 1H), 5.17 (br s, 1H), 5.08 (br s, 1H), 4.02 (t, *^3^J* = 6.9 Hz, 2H), 3.58–3.54 (m, 1H), 3.15–3.12 (m, 4H), 2.91–2.83 (m, 6H), 2.57–2.56 (m, 2H), 2.14–2.10 (m, 2H), 1.80–1.78 (m, 2H), 1.39–1.35 (m, 22H). Q-DEPT NMR (101 MHz, chloroform-*d*): *δ* 159.0 (np), 157.2 (np), 156.3 (np), 147.0 (np), 127.4, 114.3, 107.1, 79.7 (np, 2C), 56.9 (np), 54.4 (np, 2C), 51.1, 50.2 (np), 48.4 (np, 2C), 44.7 (np), 30.1 (np), 28.6 (6C), 27.3 (np), 25.5 (np), 21.2 (np). HRMS (ESI): *m/z* calculated for C_27_H_48_N_5_O_6_, [M + H]^+^ 538.3605, found 538.3611.

*N*-(3,4-diaminobutyl)-2-(3-hydroxy-2-methyl-4-oxopyridin-1(4*H*)-yl)acetamide dihydrochloride (**Ia-3**). Compound **Ia-3** was obtained from compound **26a** (0.2 mmol) according to the general procedure (Pd/C, 48 h) as a colorless oil (82 mg, 100%). *ν*_max_: 3335, 3265, 3210, 3112, 2941, 1658, 1631, 1556, 1500, 1379, 1247, 639 cm^−1^. ^1^H NMR (400 MHz, methanol-*d_4_*): *δ* 7.54 (d, *^3^J* = 7.0 Hz, 1H), 6.39 (d, *^3^J* = 7.2 Hz, 1H), 4.83–4.75 (m, 2H), 3.40–3.14 (m, 3H), 2.94–2.53 (m, 2H), 2.31 (s, 3H), 1.75–1.47 (m, 2H). Q-DEPT NMR (101 MHz, methanol-*d_4_*): *δ* 171.3 (np), 168.8 (np), 147.0 (np), 140.3, 133.5 (np), 112.6, 56.9 (np), 50.5, 39.1 (np), 37.6 (np), 35.7 (np), 12.1. HRMS (ESI): *m/z* calculated for C_12_H_21_N_4_O_3_, [M + H]^+^ 269.1614, found 269.1617. LCMS: tr 0.9 min (98% A/2% B), 190 nm: purity > 86%.

*N*-(3,4-diaminobutyl)-3-(3-hydroxy-2-methyl-4-oxopyridin-1(4*H*)-yl)propanamide dihydrochloride (*Ia-4*). Compound **Ia-4** was obtained from compound **26b** (0.4 mmol) according to the general procedure (Pd(OH)_2_/C, 48 h) as a white pasty solid (104 mg, 98%). *ν*_max_: 3322, 3244, 3073, 2927, 1626, 1555, 1500, 1353, 1238, 824, 613 cm^−1^. ^1^H NMR (400 MHz, methanol-*d_4_*): *δ* 7.52 (d, *^3^J* = 7.2 Hz, 1H), 6.32 (d, *^3^J* = 7.2 Hz, 1H), 4.30 (t, *^3^J* = 6.5 Hz, 2H), 3.30–3.12 (m, 3H), 2.63–2.59 (m, 2H), 2.53–2.44 (m, 2H), 2.42 (s, 3H), 1.54–1.25 (m, 2H). Q-DEPT NMR (101 MHz, methanol-*d_4_*): *δ* 171.8 (np), 171.0 (np), 147.8 (np), 139.0, 132.9 (np), 112.7, 51.3, 48.1 (np), 37.7 (np), 37.1 (np), 35.4 (np), 12.0. HRMS (ESI): *m/z* calculated for C_13_H_23_N_4_O_3_, [M + H]^+^ 283.1770, found 283.1762. LCMS: tr 0.9 min (98% A/2% B), 190 nm: purity >77%.

#### 3.2.8. Final *N*,*N*’-di-Boc Removal of Compounds **25a-e** and **28a-e**

General procedure: To a solution of compound **25a-e** or **28a-e** (0.1–0.4 mmol) dissolved in 1,4–dioxane (2–5 mL) was added a 4 N HCl solution (20 equiv) in 1,4-dioxane. The mixture was stirred at rt for 2–48 h and concentrated under reduced pressure. The residue was washed with Et_2_O (2 × 2–5 mL) that was then evaporated in vacuo to give compound **Ia-1**, **Ia-2**, **Ib-1**, **Ib-2**, **IIa-1** to **IIa-4** or **IIb-1** as precipitate.

*N*-(4,5-diaminopentyl)-2-(3-hydroxy-2-methyl-4-oxopyridin-1(4*H*)-yl)acetamide dihydrochloride (**Ia-1**). Compound **Ia-1** was obtained from compound **25a** (0.2 mmol) according to the general procedure (4 h) as a white solid (99 mg, 100%). mp 249 °C. *ν*_max_: 3299, 3205, 3056, 2929, 2866, 1678, 1568, 1508, 1340, 1270, 1039, 833, 633 cm^−1^. ^1^H NMR (400 MHz, methanol-*d_4_*): *δ* 8.22 (d, *^3^J* = 5.7 Hz, 1H), 7.16 (d, *^3^J* = 5.8 Hz, 1H), 5.29 (s, 2H), 3.66–3.61 (m, 1H), 3.37–3.32 (m, 4H), 2.54 (s, 3H), 1.95–1.60 (m, 4H). Q-DEPT NMR (400 MHz, methanol-*d_4_*): *δ* 167.1 (np), 160.4 (np), 144.8 (np), 144.0 (np), 149.0, 111.4, 59.1 (np), 50.6, 42.1 (np), 39.9 (np), 28.9 (np), 25.8 (np), 13.1. HRMS (ESI): *m/z* calculated for C_13_H_23_N_4_O_3_, [M + H]^+^ 283.1770, found 283.1772. LCMS: tr 0.8 min (98% A/2% B), 190 nm: purity >99%.

*N*-(5,6-diaminohexyl)-2-(3-hydroxy-2-methyl-4-oxopyridin-1(4*H*)-yl)acetamide dihydrochloride (**Ia-2**). Compound **Ia-2** was obtained from compound **25b** (0.2 mmol) according to the general procedure (48 h) as a white pasty solid (76 mg, 100%). *ν*_max_: 3214, 3044, 2917, 2856, 1667, 1632, 1502, 1338, 1254, 1117, 1034, 869, 614 cm^−1^. ^1^H NMR (400 MHz, methanol-*d_4_*): *δ* 8.19 (d, *^3^J* = 6.7 Hz, 1H), 7.16 (d, *^3^J* = 6.6 Hz, 1H), 5.26 (s, 2H), 3.62–3.60 (m, 1H), 3.34–3.29 (m, 4H), 2.52 (s, 3H), 1.90–1.72 (m, 2H), 1.66–1.61 (m, 2H), 1.59–1.53 (m, 2H). Q-DEPT NMR (400 MHz, methanol-*d_4_*): *δ* 166.9 (np), 160.4 (np), 144.8 (np), 144.0 (np), 140. 9, 111.4, 59.2 (np), 50.9, 42.1 (np), 40.2 (np), 31.1 (np), 29.7 (np), 23.2 (np), 13.1. HRMS (ESI): *m/z* calculated for C_14_H_25_N_4_O_3_, [M + H]^+^ 297.1927, found 297.1913. LCMS: tr 0.9 min (98% A/2% B), 190 nm: purity > 99%.

1-(2-(4-(4,5-diaminopentanoyl)piperazin-1-yl)-2-oxoethyl)-3-hydroxy-2-methylpyridin-4(1*H*)-one dihydrochloride (**Ib-1**). Compound **Ib-1** was obtained from compound **25c** (0.3 mmol) according to the general procedure (36 h) as a white solid (158 mg, 100%). mp 177 °C. *ν*_max_: 3289, 2909, 2854, 2655, 1628, 1441, 1336, 1224, 1117, 1018, 869, 812 cm^−1^. ^1^H NMR (400 MHz, water-*d_2_*): *δ* 7.96 (d, *^3^J* = 6.8 Hz, 1H), 7.15 (d, *^3^J* = 6.9 Hz, 1H), 5.54 (s, 2H), 3.85–3.76 (m, 3H), 3.73–3.69 (m, 6H), 3.42–3.40 (m, 2H), 2.81–2.76 (m, 2H), 2.48 (s, 3H), 2.18–2.05 (m, 2H). Q-DEPT NMR (101 MHz, water-*d_2_*): *δ* 172.4 (np), 165.2 (np), 159.4 (np), 143.4 (np), 142.5 (np), 139.9, 111.0, 57.3 (np), 48.9, 44.6 (np), 44.2 (np), 42.1 (np), 41.3 (np), 40.7 (np), 28.4 (np), 25.3 (np), 12.5. HRMS (ESI): *m/z* calculated for C_17_H_28_N_5_O_4_, [M + H]^+^ 366.2141, found 366.2133. LCMS: tr 0.9 min (98% A/2% B), 190 nm: purity >71%.

1-(2-(4-(4,5-diaminopentanoyl)piperazin-1-yl)-2-oxoethyl)-3-hydroxypyridin-2(1*H*)-one dihydrochloride (**Ib-2**). Compound **Ib-2** was obtained from compound **28a** (0.3 mmol) according to the general procedure (6 h) as an off-white solid (181 mg, 100%). mp 181 °C. *ν*_max_: 3034, 2917, 2853, 1586, 1442, 1216, 1117, 1014, 870, 618 cm^−1^. ^1^H NMR (400 MHz, water-*d_2_*): *δ* 7.13–7.07 (m, 2H), 6.46–6.42 (m, 1H), 5.01 (s, 2H), 3.94–3.86 (m, 1H), 3.72–3.52 (m, 8H), 3.34–3.17 (m, 2H), 2.77–2.75 (m, 2H), 2.12–2.07 (m, 2H). Q-DEPT NMR (101 MHz, water-*d_2_*): *δ* 172.3 (np), 167.1 (np), 158.8 (np), 145.4 (np), 130.1, 119.2, 108.3, 51.1 (np), 48.9, 44.6 (np), 44.2 (np), 41.8 (np), 41.4 (np), 40.7 (np), 28.4 (np), 25.3 (np). HRMS (ESI): *m/z* calculated for C_16_H_26_N_5_O_4_, [M + H]^+^ 352,1985, found 352,1981. LCMS: tr 1.0 min (98% A/2% B), 190 nm: purity > 84%.

1-(5,6-diaminohexyl)-3-hydroxy-2-methylpyridin-4(1*H*)-one dihydrochloride (**IIa-1**). Compound **IIa-1** was obtained from compound **25d** (0.4 mmol) according to the general procedure (3 h) as a off-white pasty solid (110 mg, 86%). *ν*_max_: 3355, 2973, 2932, 1633, 1509, 1339, 1249, 1163, 1048 cm^−1^. ^1^H NMR (400 MHz, methanol-*d_4_*): *δ* 8.25 (d, *^3^J* = 7.0 Hz, 1H), 7.13 (d, *^3^J* = 6.9 Hz, 1H), 4.42 (t, *^3^J* = 7.6 Hz, 2H), 3.68–3.61 (m, 1H), 3.31–3.27 (m, 2H), 2.64 (s, 3H), 1.96–1.77 (m, 4H), 1.62–1.54 (m, 2H). Q-DEPT NMR (101 MHz, methanol-*d_4_*): *δ* 159.5 (np), 145.0 (np), 143.1 (np), 139.3, 111.8, 57.4, 50.6, 42.1, 31.0, 30.7, 22.7, 12.9. HRMS (ESI): *m/z* calculated for C_12_H_22_N_3_O_2_, [M + H]^+^ 240.1712, found 240.1713. LCMS: tr 0.8 min (98% A/2% B), 190 nm: purity >99%.

1-(10,11-diaminoundecyl)-3-hydroxy-2-methylpyridin-4(1*H*)-one dihydrochloride (**IIa-2**). Compound **IIa-2** was obtained from compound **25e** (0.1 mmol) according to the general procedure (18 h) as a brown pasty solid (23 mg, 100%). *ν*_max_: 3373, 2925, 2855, 1630, 1506, 1336, 1257, 1032, 715, 635 cm^−1^. ^1^H NMR (400 MHz, water-*d_2_*): *δ* 8.05 (m, 1H), 7.12 (m, 1H), 4.35–4.31 (m, 2H), 3.65-3.61 (m, 1H), 3.35–3.34 (m, 2H), 2.61 (s, 3H), 1.85–1.67 (br s, 4H), 1.46–1.42 (m, 2H), 1.36–1.32 (m, 14H). ^13^C NMR (101 MHz, water-*d_2_*): *δ* 158.5, 142.2, 138.4, 120.0, 110.9, 56.8, 49.4, 40.7, 30.0, 29.4, 28.4, 28.3 (2C), 26.5, 25.3, 24.0, 12.2. HRMS (ESI): *m/z* calculated for C_17_H_32_N_3_O_2_, [M + H]^+^ 310.2495, found 310.2490. LCMS: tr 2.8 min (98% A/2% B), 190 nm: purity >99%.

1-(5,6-diaminohexyl)-3-hydroxypyridin-2(1*H*)-one dihydrochloride (**IIa-3**). Compound **IIa-3** was obtained from compound **28c** (0.1 mmol) according to the general procedure (18 h) as a brown pasty solid (8 mg, 60%). *ν*_max_: 3588, 3380, 1648, 1557, 1541, 673 cm^−1^. ^1^H NMR (400 MHz, methanol-*d_4_*): *δ* 7.30–7.23 (m, 1H), 6.97–6.93 (m, 1H), 6.46–6.36 (m, 1H), 4.19–4.14 (m, 2H), 3.63–3.59 (m, 1H), 3.30–3.24 (m, 2H), 1.99–1.76 (m, 4H), 1.57–1.45 (m, 2H). HRMS (ESI): *m/z* calculated for C_11_H_20_N_3_O_2_, [M + H]^+^ 226.1556, found 226.1557. LCMS: tr 0.8 min (98% A/2% B), 190 nm: purity >89%.

1-(10,11-diaminoundecyl)-3-hydroxypyridin-2(1*H*)-one dihydrochloride (**IIa-4**). Compound **IIa-4** was obtained from compound **28d** (0.1 mmol) according to the general procedure (18 h) as a brown pasty solid (28 mg, 74%). *ν*_max_: 3375, 2923, 2852, 1646, 1579, 1461, 1258, 1049, 750, 645cm^−1^. ^1^H NMR (400 MHz, water-*d_2_*): *δ* 7.24–7.20 (m, 1H), 7.07–7.03 (m, 1H), 6.44–6.40 (m, 1H), 4.03–3.99 (m, 2H), 3.66–3.62 (m, 1H), 3.38–3.34 (m, 2H), 1.75–1.71 (br s, 4H), 1.44–1.40 (m, 2H), 1.31–1.27 (m, 14H). ^13^C NMR (101 MHz, water-*d_2_*): *δ* 158.4, 145.3, 129.5, 118.5, 108.3, 50.4, 49.4, 40.8, 29.9, 28.5, 28.4, 28.3, 28.2, 28.1, 25.6, 24.0. HRMS (ESI): *m/z* calculated for C_16_H_30_N_3_O_2_, [M + H]^+^ 296.2338, found 296.2338. LCMS: tr 0.9 min (98% A/2% B), 190 nm: purity >91%.

1-(3-(4-(4,5-diaminopentyl)piperazin-1-yl)propyl)-3-hydroxypyridin-2(1*H*)-one dihydrochloride (**IIb-1**). Compound **IIb-1** was obtained from compound **28e** (0.3 mmol) according to the general procedure (2 h) as a off-white pasty solid (120 mg, 78%). *ν*_max_: 3365, 2924, 2852, 1647, 1537, 1270, 1068, 752 cm^−1^. ^1^H NMR (400 MHz, dimethylsulfoxide-*d_6_*): *δ* 8.77 (br s, 4H), 7.21 (d, *^3^J* = 6.5 Hz, 1H), 6.72 (dd, *^3^J* = 7.2, 1.7 Hz, 1H), 6.13 (t, *^3^J* = 7.0 Hz, 1H), 4.47–4.11 (m, 2H), 4.04–4.01 (m, 2H), 3.89–3.66 (m, 4H), 3.51–3.41 (m, 4H), 3.39–3.32 (m, 1H), 3.19–3.04 (m, 4H), 2.14–2.11 (m, 2H), 1.97–1.83 (m, 2H), 1.76–1.67 (m, 2H). Q-DEPT NMR (101 MHz, dimethylsulfoxide-*d_6_*): *δ* 157.8 (np), 146.8 (np), 128.0, 115.0, 105.8, 50.2, 46.3 (np, 4C), 46.0 (np, 2C), 39.6 (np, 2C), 25.8 (np), 23.3 (np), 18.8 (np). HRMS (ESI): *m/z* calculated for C_17_H_32_N_5_O_2_, [M + H]^+^ 338.2556, found 338.2552. LCMS: tr 0.9 min (98% A/2% B), 190 nm: purity >86%.

### 3.3. Physicochemical Evaluations

#### 3.3.1. Oxygen Radical Absorbance Capacity (ORAC) Assay

A fluorescein (FL) solution (12 nM, 150 µL) was introduced in a black 96-well plate (Dutscher, Brumath, France). Then, tested compounds (0.1–20 μM, 25 μL) or 6-hydroxy-2,5,7,8-tetramethylchroman-2-carboxylic acid (Trolox) standard (1–50 μM, 25 μL) dissolved in D-PBS were added to each well. The plate was allowed to equilibrate by incubating for a minimum of 30 min at 37 °C. Reactions were initiated by the addition of a freshly prepared 2,2’-azobis(2-methylpropionamidine) dihydrochloride (AAPH) solution (30 mM, 25 µL). The fluorescence (λ_Ex_: 485 nm; λ_Em_: 520 nm) was measured using a thermostated Tecan Infinite^®^ 200 PRO microplate reader every 90 s for 60 cycles. All reaction mixtures were prepared in triplicate, and at least three independent assays were performed for each sample. FL fluorescence decay curve in time allowed measuring AUC of tested products in comparison with the control corresponding to the absence of an antioxidant. ORAC_FL_ values at 10 µM were calculated with respect to the linear equation of trolox calibration curve (Net AUC vs. concentration) and expressed as µmol trolox equivalent (TE)/µmol of tested compound.

#### 3.3.2. RCS Trapping Assay

Aqueous 40% MGO solution was diluted in water (200 mM, 1.25 mL) and MDA solution (200 mM, 1.25 mL) was freshly prepared by hydrolyzing malonaldehyde bis(diethyl acetal) (250 µmol) with 1 N HCl (2 equiv) at rt for 1 h under vigorous stirring. A stock solution of tested compounds (30 µmol) was also prepared in D-PBS (20 mM, 1.5 mL). Tested compounds (10 mM) were incubated with MGO or MDA (20 mM) at 37 °C for 24 h. The pH of the solution was adjusted to 7.4 using 0.02 N NaOH (final mixture volume, 1.25 mL). Samples (100 µL) were collected at regular time intervals (0.25, 0.5, 1, 5 and 24 h) and stored at −20 °C to stop the reaction. After leaving to rt, they were submitted to LC-LRMS analysis (generally, tr_Free scavengers_: 0.8–1.3 min, tr_Adducts_: 5.1–5.5 min). A blank solution without scavenger and a standard one with only scavenger were used as well. After identification of unreacted free scavenger and adducts on mass spectrum, AUC of total peak of adducts and remaining free scavenger peak were measured on a UV chromatogram to give the % of adduct formation.

#### 3.3.3. Cu^2+^-Chelating Assay

Stock solutions of tested compounds (4.2 mM, 6.2 mL) and CuSO_4._5H_2_O (0.25 mM, 100 mL for the assay and 0.5 mM, 20 mL for the calibration curve) were prepared in 10 mM hexamine/HCl buffer containing 10 mM KCl (pH 5.0) as well as aqueous solution of murexide (1 mM, 10 mL). Tested compounds (0.02, 0.05, 0.08, 0.1, 0.2, 0.5, 1 and 2 mM) were diluted in buffer (Q.S. to 1 mL) and incubated with CuSO_4._5H_2_O (120 µM, 1 mL) at rt for 10 min. Murexide (48 µM, 0.1 mL), used as complexometric indicator was then added and the mixture was incubated for another 1 min at rt. Absorbance of the solution was recorded at 485 nm (λ_max_ of Cu^2+^/murexide complex) and 520 nm (λ_max_ of free murexide) using a Jasco V-650 UV/Vis spectrophotometer. A blank solution composed of buffer (2 mL) and water (0.1 mL) was required. Calibration curves (A_485_/A_520_ vs. Cu^2+^ concentration) were previously plotted using CuSO_4._5H_2_O (0, 25, 50, 75, 100 and 125 µM) in buffer (Q.S. to 2 mL) and murexide solution (0.1 mL). Knowing the total quantity of metal ions introduced into the reaction mixture (control conditions without tested product), % Cu^2+^ chelation by tested compounds was estimated by difference.

### 3.4. Biological Evaluation

#### 3.4.1. Cell Viability

The immortalized rat neuronal-like cell line PC12 Adh (CRL-1721.1, LGC, Molsheim, France) was derived from primary rat pheochromocytoma. PC12 cells were cultured in F-12K medium supplemented with 2.5% heat-inactivated fetal bovine serum (FBS, PAN biotech., Dutscher, Brumath, France), 15% heat-inactivated horse serum (HS, Life technologies SAS, Saint-Aubin, France), 100 IU/mL penicillin and 100 µg/mL streptomycin at 37 °C in humidified atmosphere containing 5% CO_2._

Cell viability was assessed by measuring mitochondrial activity in the presence of 2-(2-methoxy-4-nitrophenyl)-3-(4-nitrophenyl)-5-(2,4-disulfophenyl)-2*H*-tetrazolium, monosodium salt (WST-8), that produces a water-soluble formazan dye upon bio-reduction in the presence of an electron carrier, 1-methoxy-5-methylphenazinium methyl sulphate (1-Methoxy PMS). Briefly, cells (5.10^3^ cells/well) were seeded in 96-well plates (Corning, VWR, Fontenay-sous-bois, France) in 100 µL of appropriate complete medium. Once cells have reached 80% confluency, the medium was changed and cells were treated with various concentrations of tested compounds (10 µM or 100 µM) for 24 h. A positive control of cytotoxicity was used by adding 10% DMSO. The CCK8 solution (10 µL) was directly added to each well and incubated for another 1 h at 37 °C. Absorbance was recorded at 450 nm using a Perkin Elmer 2103 Envision^®^ multilabel microplate reader and provided an estimation of formazan dye bioproduction that was directly proportional to the number of living cells. Cell viability was expressed as % of control (non-treated cells) and at least three independent experiments were performed in triplicate.

#### 3.4.2. *In Vitro* MGO-Induced Apoptosis Inhibition in the Model AD Cell-Line PC12

MGO-induced apoptosis was measured by the detection of DNA fragmentation. Briefly, cells were pre-incubated with tested compounds (10 µM or 100 µM) dissolved in D-PBS for 1 h at 37 °C before adding MGO (1 mM) for another 24 h incubation. Apoptosis was evaluated by an ELISA assay for cytoplasmic histone-associated DNA fragments (mono- and oligonucleosomes), using a cell death detection kit from Roche Diagnostics (Meylan, France). Cell lysates were applied to a plate coated with an anti-histone antibody. A peroxidase labeled anti-DNA antibody was then added and detected with 2,2′-azino-bis(3-ethylbenzothiazoline-6-sulfonic acid) diammonium salt (ABTS) as a substrate. Reacting with hydrogen peroxide, it turned into a green soluble end-product whose optical density (OD) reflecting apoptosis level was read at 405 nm using a Perkin Elmer 2103 Envision^®^ multilabel microplate reader. Cells incubated without MGO represented control conditions and a positive apoptosis standard was assessed in the presence of MGO alone.

## 4. Conclusions

In continuation of our preceding studies [8,9], two novel series Ia-b and IIa-b of hydroxypyridinone-diamine hybrids have been developed as multifunctional scavengers to thwart AD-triggering biometal-mediated vicious downward redox spiral. Eleven new final products have been synthesized in 7–11 steps and 2%–57% overall yields. This extended work allowed us to highlight the new lead compound IIa-3 bearing a C4 alkyl linker between the two pharmacophores. This derivative revealed preserved potent RCS, ROS and Cu^2+^-trapping capacities in comparison with previously described lead compound 1. In addition, the improvements of its Qikprop-predicted lipophilicity and ability to cross the blood brain barrier were correlated with a better efficacy to inhibit *in vitro* MGO-induced apoptosis in neuronal-like PC12 cells.

This new AGE/ALE inhibitor IIa-3 seems to be able to prevent biometal-mediated oxidative and carbonyl stress progression in this PC12 cell-line model. Further *in vitro* and in vivo biological investigations are required to confirm its therapeutic potential in Alzheimer ‘s disease.

## 5. Patents

The WIPO Patent Application WO 2017/006048 A1 is the result of the work reported in this manuscript.

## Figures and Tables

**Table 1:** Diamine building blocks and HOPO ligands (or other related precursors) implicated in the key coupling step of our AGE/ALE inhibitor synthetic strategy.

Diamine Building Blocks ^1^	HOPO Ligands ^1,2^
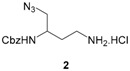	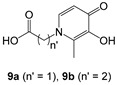
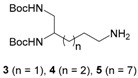	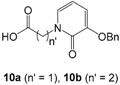
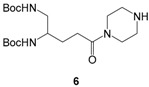	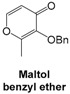
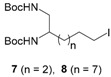	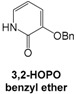

^1^ Diamine building blocks **3**, **4** and **6** as well as HOPO ligands **9b** and **10b** have been described in our previous publication [8]. ^2^ HOPO ligands **9a** and **10a** as well as 3,2-HOPO and maltol benzyl ethers have been previously described in the literature [10,11,12,13].

**Table 2:** QikProp-predicted absorption, distribution, metabolism and excretion (ADME) properties of HOPO-diamine hybrids as drug candidates.

Series	Linker	HOPO Moiety	Compound	logP_o/w_^1^	logBB ^2^	P_Caco_^3^	P_MDCK_^4^	CNS Activity ^5^
**Ia**	amide	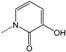	**1**	**−1.88**	**−1.77**	**1.73**	**1.07**	**−2**
		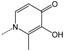	**Ia-1**	−1.86	−1.34	1.99	1.30	−2
			**Ia-2**	−1.54	−1.54	2.16	1.34	−2
			**Ia-3**	−2.10	−1.17	2.40	1.50	−2
			**Ia-4**	−1.42	−1.35	3.70	2.12	−2
**Ib**	piperazine-1,4-diamide	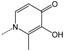	**Ib-1**	−2.72	−1.90	0.34	0.44	−2
		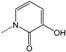	**Ib-2**	−2.80	−1.82	0.77	0.74	−2
			**Ib-3**	−2.52	−1.63	1.05	1.01	−2
**IIa**	alkyl	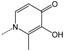	**IIa-1**	−0.58	−1.03	7.04	2.86	−2
			**IIa-2**	0.98	−1.60	6.70	2.71	−2
		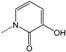	**IIa-3**	**−0.82**	**−0.86**	**10.21**	**4.27**	**−1**
			**IIa-4**	0.71	−1.44	9.65	4.02	−2
**IIb**	piperazine-1,4-dialkyl	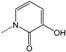	**IIb-1**	−1.31	−1.41	1.92	0.78	−2

^1^ P_o/w_ represent the octanol/water partition coefficient. QikProp-recommended values for logP_o/w_ are ranged from −2 to 6.5. ^2^ BB represent the brain/blood partition coefficient for orally delivered drugs. QikProp-recommended values for logBB are ranged from −3 to 1.2. ^3^ P_Caco_ represent the apparent Caco-2 cell permeability in nm/sec. Caco-2 cells constitute a gutblood barrier model. QikProp-recommended P_Caco_ values are significant for non-active transport. P_Caco_ values <25 correspond to a poor permeability whereas P_Caco_ values >500 correspond to a great permeability. ^4^ P_MDCK_ represent the apparent MDCK cell permeability in nm/sec. MDCK cells constitute a blood–brain barrier model. QikProp-recommended P_MDCK_ values are significant for non-active transport. P_Caco_ values <25 correspond to a poor permeability whereas P_MDCK_ values >500 correspond to a great permeability. ^5^ CNS activity represent the QikProp-predicted central nervous system activity of drug candidate on a −2 (inactive) to +2 (active) scale.

**Table 3:** Reactive carbonyl species (RCS) trapping, oxygen radical absorbance and Cu^2+^-chelating capacities of new HOPO-diamine hybrids in comparison with previous lead compound **1** and references.

Compound	% MGO Adduct Formation ^1^	% MDA Adduct Formation ^2^	ORAC_FL_^3^ (µmol TE/µmol)	% Cu^2+^ Chelation ^4^
**Trolox**	ND	ND	1	ND
**EDA**	ND	ND	ND	100 *
**Carnosine**	46 *	40 *	0.08 ± 0.05 *	1.53 ± 1.18 *
**1**	97 *	81 *	1.96 ± 0.24 *	55.83 ± 0.17 *
**Ia-1**	ND	ND	1.03 ± 0.11	ND
**Ia-2**	ND	ND	0.90 ± 0.09	ND
**Ia-3**	79	83	0.81 ± 0.08	89.45 ± 0.35
**Ia-4**	ND	ND	1.67 ± 0.16	ND
**Ib-1**	ND	ND	0.53 ± 0.13	ND
**Ib-2**	ND	ND	0.86 ± 0.12	ND
**Ib-3**	97 *	81 *	0.99 ± 0.09 *	53.23 ± 0.40 *
**IIa-1**	ND	ND	0.88 ± 0.01	ND
**IIa-2**	ND	ND	ND	ND
**IIa-3**	**100**	**40**	**0.98 ± 0.03**	**60.11 ± 0.83**
**IIa-4**	ND	ND	0.84 ± 0.04	ND
**IIb-1**	ND	ND	0.57 ± 0.03	ND

^1^ Data obtained in 15 min of incubation at 37 °C for a representative single sample. ^2^ Data obtained in 1 h of incubation at 37 °C for a representative single sample. ^3^ ORAC_FL_ values are expressed as means ± standard error of the mean (SEM) of three independent experiments performed in triplicate and calculated at 10 µM. ^4^ % Cu^2+^ chelation are expressed as means ± SEM of triplicates and measured at 200 µM. * Previously reported data [8]; TE: Trolox equivalent; ND: Not determined.
